# Understanding the distribution and fine-scale habitat selection of mesocarnivores along a habitat quality gradient in western Himalaya

**DOI:** 10.7717/peerj.13993

**Published:** 2022-09-16

**Authors:** Meghna Bandyopadhyay, A. Cole Burton, Sandeep Kumar Gupta, Ramesh Krishnamurthy

**Affiliations:** 1 Wildlife Institute of India, Dehradun, Uttarakhand, India; 2 Faculty of Forestry, University of British Columbia, Vancouver, Canada

**Keywords:** Modified habitat, Human-wildlife interface, Red fox, Leopard cat, Camera trap, Rugged terrain, Genetic approach, Great Himalayan National Park, Habitat preference, Hierarchical generalized additive modelling

## Abstract

**Background: **Human activities have resulted in a rapid increase of modified habitats in proximity to wildlife habitats in the Himalaya. However, it is crucial to understand the extent to which human habitat modification affects wildlife. Mesocarnivores generally possess broader niches than large carnivores and adapt quickly to human activities. Here, we use a case study in the western Himalaya to test the hypothesis that human disturbance influenced mesocarnivore habitat use.

**Methods: **We used camera trapping and mitochondrial DNA-based species identification from faecal samples to obtain mesocarnivore detections. We then compared the responses of mesocarnivores between an anthropogenic site and a less disturbed park along a contiguous gradient in habitat quality. The non-linear pattern in species-specific habitat selection and factors responsible for space usage around villages was captured using hierarchical generalized additive modelling (HGAM) and non-metric multidimensional scaling (NMDS) ordination.

**Results: **Wildlife occurrences along the gradient varied by species. Leopard cat and red fox were the only terrestrial mesocarnivores that occurred in both anthropogenic site and park. We found a shift in habitat selection from less disturbed habitat in the park to disturbed habitat in anthropogenic site for the species detected in both the habitat types. For instance, red fox showed habitat selection towards high terrain ruggedness (0.5 to 0.7 TRI) and low NDVI (−0.05 to 0.2) in the park but no such specific selection in anthropogenic site. Further, leopard cat showed habitat selection towards moderate slope (20°) and medium NDVI (0.5) in park but no prominent habitat selections in anthropogenic site. The results revealed their constrained behaviour which was further supported by the intensive site usage close to houses, agricultural fields and human trails in villages.

**Conclusions: **Our results indicate shifts in habitat selection and intensive site usage by mesocarnivores in the human-modified habitat. In future, this suggests the possibility of conflict and disease spread affecting both the people and wildlife. Therefore, this study highlights the requisite to test the wildlife responses to rapidly growing human expansions in modified habitats to understand the extent of impact. The management strategies need to have an integrated focus for further expansions of modified habitat and garbage disposal strategies, especially in the human-wildlife interface area.

## Introduction

Human settlement is one of the most substantial factors modifying habitat conditions for wildlife worldwide (Vitousek et al., 1997; Andersen et al., 2017). The encroachment of human settlements into surrounding ecosystems creates new ecological niches (Mckinney, 2002) and also alters existing niches. As a result, there are opportunities for an increase in anthropogenic food sources for wildlife in and around the human settlements (Verdade et al., 2011). These food sources benefit some species by affecting their behaviour, distributions and interspecific interactions (Parmesan, 2006). For instance, coyotes and white-tailed deer were considered sensitive to human activities but have colonised urban landscapes in recent decades (Ditchkoff, Saalfeld & Gibson, 2006). Further, a wide range of wild carnivores inhabited the cropland landscape (Athreya et al., 2013) and agroecosystem matrix (Ferreira et al., 2018). Some carnivores adapt quickly to human-modified habitats by utilising anthropogenic food sources (Ghoshal, 2011; Athreya et al., 2016; Naha et al., 2020b), while some are adversely affected by these habitat alterations (Carricondo-Sanchez et al., 2019).

The Himalaya has faced consistent pressure from increased human settlements due to agricultural practices, more intensive grazing by domestic animals and increased demand for timber (Cronin, 1979; Schaller, 1980). Consequently, the increasing quantity and proximity of readily available anthropogenic subsidies facilitate increasing dependencies of native carnivores on these resources (Ghoshal, 2011; Ghoshal et al., 2016; Rajaratnam, Vernes & Sangay, 2016; Khan et al., 2020). Additionally, the natural resources are localised in the rugged landscapes and often are not readily available to carnivores compared to the anthropogenic food resources. Moreover, when anthropogenic food resources replace natural prey due to habitat modifications, such changes affect wildlife distribution (Ripple et al., 2014; Parsons, Newsome & Young, 2022). In this context, there is a paucity of information regarding the modified habitats adjacent to less disturbed natural forests and their effect on the native wildlife in the rugged landscape of Himalaya.

In this study, we studied mesocarnivores to understand the impact of habitat modification in the western Himalaya. Mesocarnivores are known for their diverse behaviour and ecology; hence they are more generalist when living in close proximity to humans than large carnivores (Roemer, Gompper & Van Valkenburgh, 2009). Their diverse nature makes them receptive to small-scale habitat alterations, and they responds more quickly than large carnivores (Randa & Yunger, 2006). Thus, they serve as helpful indicator species in preserving sensitive habitats (Kalle et al., 2013; Torre et al., 2022). Apart from being an indicator species, mesocarnivores efficiently utilise anthropogenic food sources, like garbage dumps, agricultural products, kitchen wastes and livestock carrions (Reshamwala et al., 2018), due to their opportunistic behaviour and ability to adapt to modified habitats (Rajaratnam et al., 2007; Lorica & Heaney, 2013). In this situation, the site usage near human habitations by mesocarnivores enables the shared spaces by wildlife and human to be the most probable zone for zoonotic disease spread (Ghimire, Regmi & Huettmann, 2020) and conflict risks (Peterson et al., 2021). Mesocarnivores are known to be the potential hosts of zoonoses at the wildlife-livestock-human interface (Yang et al., 2021). That is why transmission of diseases like rabies, canine distemper viruses and anthrax can occur bi-directionally, affecting both humans and wildlife (Beineke, Baumgärtner & Wohlsein, 2015; Muturi et al., 2018; Acharya et al., 2020; Gonzálvez, Martínez-Carrasco & Moleón, 2021). Therefore, knowledge of mesocarnivore space usage in human-modified habitats can aid in improving management strategies in the light of future outbreaks of zoonotic diseases and conflict probabilities (Alexander et al., 2012; Theimer et al., 2017; Ng et al., 2019; Ferreira et al., 2021; Veals et al., 2021).

The Great Himalayan National Park Conservation Area (GHNPCA) of Western Himalaya comprises of heterogeneous habitat gradient. Hence, we considered it as the study site to understand the responses of mesocarnivores to habitat modification in ecozone (henceforth, anthropogenic site) and national park (henceforth, park). Change in carnivore habitat selection from a relatively less human-disturbed to a more human-disturbed area in favour of easily available resources can be seen in a continuum of mosaic habitats (Boydston et al., 2003). Therefore, studying carnivore distribution along the habitat gradient consisting of both the human-modified and natural forest (Andersen et al., 2017) will aid in (a) understanding the status of the human-wildlife interface and (b) enabling integrated management of nature and people in the susceptible landscapes. In the context of anthropogenic habitat in GHNPCA, the human population was 15,000 from 160 villages in 1995 (Tucker, 1997), reaching about 9,000 in just six villages in 2011 (www.census2011.co.in). With the increase of human population, there were conversions of forested habitats into arable lands for agricultural practices (Tucker, 1997). Since settlements and agricultural plots generate human-induced resources (Verdade et al., 2011), there are chances that the resources are available in more quantity and close to natural habitat than before. We used 3^rd^ order habitat selection of mesocarnivores related to the usage of habitat components within the home range (Johnson, 1980). We expected mesocarnivores to show changes in habitat selection in anthropogenic site relative to park in GHNPCA. Mesocarnivores prefer fine-scale forest fragments (Červinka et al., 2011) and use a variety of habitats (Gese & Thompson, 2014). Therefore, we selected habitat variables like slope, ruggedness, elevation, normalized difference vegetation index (NDVI) and distance to woodland (riparian forest at hill base) to identify the changes in the habitat selection. The selected potential habitat variables important for mesocarnivores occurrences also aligned with other studies where elevation, terrain ruggedness, *etc*., were used as explanatory variables for red fox and leopard cat occurrences (Parsons et al., 2019; Kalle et al., 2013). Although some studies have shown the utilization of human-modified habitats by mesocarnivores in the Himalayas and other areas (Verdade et al., 2011; Lorica & Heaney, 2013; Bashir et al., 2014; Ghoshal et al., 2016), information regarding the scale of such utilization is still scarce. Hence, the study focuses on the following objectives: (1) To understand the distribution pattern of mesocarnivores along the habitat gradient; (2) to determine species-specific habitat selection of mesocarnivores in the park and anthropogenic site; and (3) to determine factors responsible for space usages by mesocarnivores in the anthropogenic site.

## Materials and Methods

### Study area

**Figure 1:**
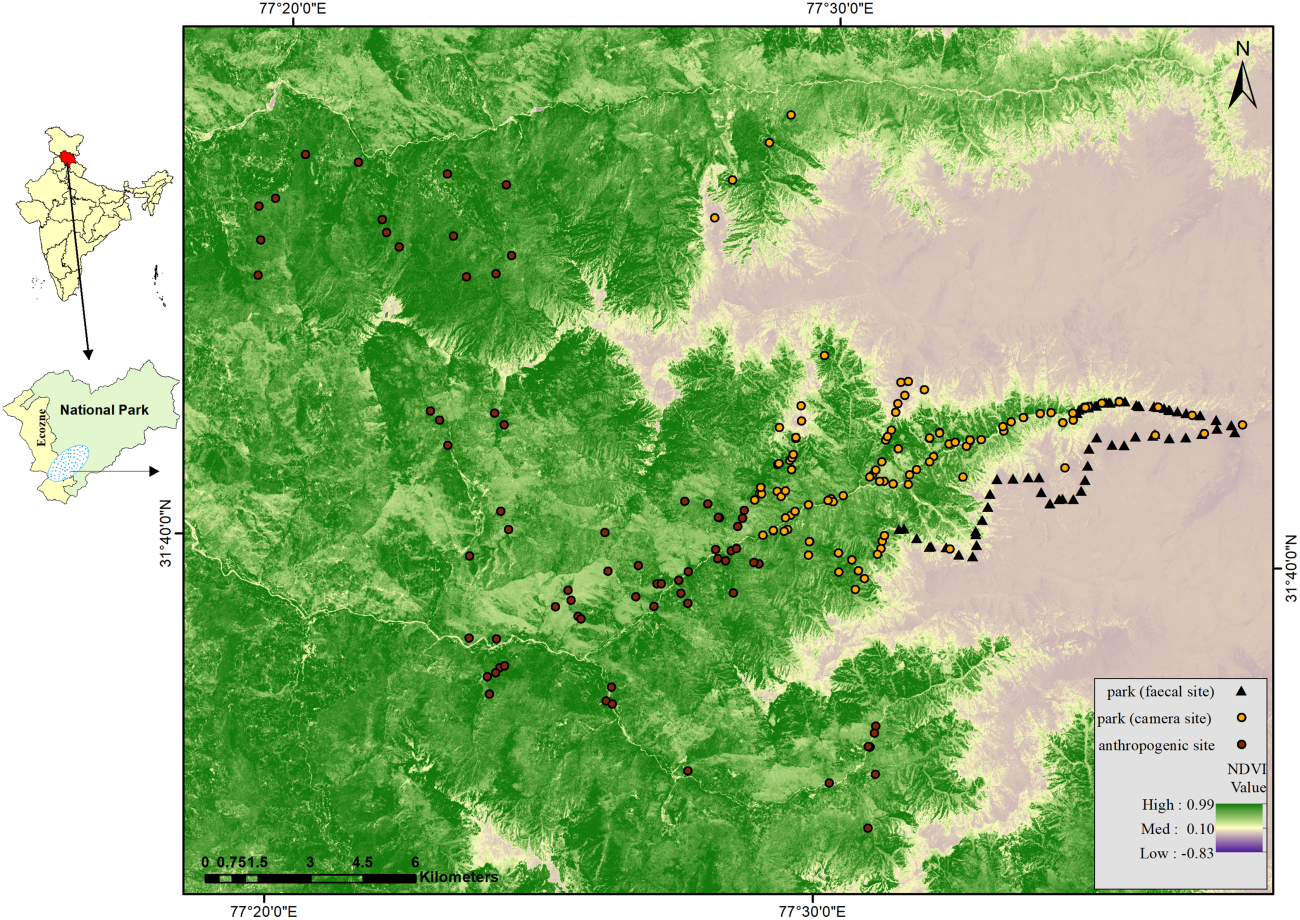
Map of GHNPCA showing sampling in park (faecal site) (survey trail mid points = 125), park (camera site) (camera trap locations = 220) and anthropogenic site (camera trap locations = 120) during 2017 to 2019.

The Great Himalayan National Park Conservation Area (GHNPCA), a UNESCO world heritage site (UNESCO, 2011; UNESCO, 2014: https://whc.unesco.org/en/list/1406/), is located in the Kullu district of Himachal Pradesh, western Himalaya, India (Fig. 1). The area of GHNPCA covers four catchments (river), viz., Parvati, Jiwa, Sainj and Tirthan. The administrative boundary divides into anthropogenic sites representing the buffer area and park representing the core forest area. We selected Tirthan for the intensive studies due to the similarity in habitat characteristics with the entire GHNPCA (Singh & Rawat, 1999). Tirthan catchment (300 sq. km.) represents a highly variegated landscape with lower temperate Chir pine (*Pinus roxburghii*), Banj oak (*Quercus leucotrichophora*) and open scrubs at lower elevation (<2,000 m) to upper temperate Fir (*Abies pindrow*), Kharsu oak (*Quercus semecarpifolia*) forests and alpine meadows at high elevation (2,500 to 4,000 m) within an aerial distance of 35 km. A detailed list of the composition of the vegetation structure is available in Singh & Rawat (1999).

### Camera trapping

We conducted camera trapping in five sessions from 2017 to 2019, covering the anthropogenic site and park in all the human-established trails. Number of sampling locations (*n*) and days of effort (t) in the respective five sessions were April–July 2017; *n* = 59, t = 2,986, October–December 2017; *n* = 78, t = 2,589, April–July 2018; *n* = 40, t = 1,791, October–December 2018; *n* = 82, t = 2,737 and April–June 2019; *n* = 81, t = 1,763. We deployed 340 camera traps from 2017 to 2019 (Fig. 1, Figs. S1 to S5) (Table S1). Total camera trap effort was 11,866 (no. of camera traps × operational days). The total number of camera traps and effort in the anthropogenic site were 120 and 2,582, and in the park, 220 and 9,284, respectively. Table S1 consists of detailed information regarding the number of days of each camera trap. We deployed camera traps systematically with a minimum distance of 0.5 km and a maximum distance of 1 km between each consecutive trap location.

### Faecal sampling

We could not conduct camera trapping and regular monitoring in some areas inside the park as the habitats have rugged terrain and harsh climatic conditions (Ramesh, Sathyakumar & Rawat, 1999; Singh & Rawat, 1999). Therefore, we adopted non-invasive methods like faecal sample collection to cover the relatively inaccessible areas inside the park for mesocarnivores showing high detections through camera trapping. Following the dry sampling protocol, we collected carnivore faecal samples opportunistically during the same period (Biswas et al., 2019). We surveyed 125 trails, each length 500 m (Fig. 1) (Table S1), and collected 161 carnivore faecal samples. We stored the samples at −20 °C and subjected them for mesocarnivore species confirmation using molecular markers (Cytochrome b, 146 bp) because of the presence of other sympatric carnivores (Vinod & Sathyakumar, 1999; Bandyopadhyay, Dasgupta & Krishnamurthi, 2019) in the study area.

### Species confirmation

We extracted DNA from faecal samples by swabbing the outer layer and following the protocol described in Ball et al. (2007) and Biswas et al. (2019). Further, we used a carnivore-specific molecular marker (Cytochrome b, 146 bp) to ascertain the species (Farrell, Roman & Sunquist, 2000). We performed PCR reactions in 10 µl reaction volumes with 5 µl multiplex master mix, 1 µl of bovine serum albumin (BSA), 0.8 µl of each primer, 0.4 µl RNAse free water and 2 µl of template DNA. PCR conditions were 95 °C for 10 min followed by 38 cycles at 95 °C for 30 s, annealing at 55 °C for 50 s and extension 72 °C for 50 s, with a final extension of 72 °C for 10 min. We monitored the effectiveness and consistency of the PCR reactions by using positive and negative controls. The amplified PCR amplicons were visualized in UV light on 2% agarose gel stained with green stain dye. Exonuclease I (EXO-I) and shrimp alkaline phosphatase (SAP) (Thermo Scientific, Waltham, MA, USA) treatments were given to the amplified PCR products for 15 min each at 37 °C and 80 °C, respectively, to eliminate any residual primer and unused dNTPs. The amplified PCR products were sequenced using the BigDye® Terminator cycle sequencing Kit (v3.1; Thermo Fisher Scientific, Waltham, MA, USA) and analyzed on an ABI 3500XL Applied Biosystems Genetic Analyzer (ABI 3500xl, Applied Biosystems, Waltham, MA, USA). Finally, we identified the sequences by comparing them in the NCBI database using the BLAST tool (http://blast.ncbi.nlm.nih.gov/Blast.cgi).

### Data analysis

#### Distribution pattern

We used 15 min as the minimum interval to consider species detection from camera traps (photo capture, C) as independent detections for estimating the relative abundance index (RAI; capture rate per 100 trap night) of mesocarnivores in the park and anthropogenic site. Red fox (*Vulpes vulpes*) and leopard cat (*Prionailurus bengalensis*) were the only terrestrial mesocarnivores detected in the park and anthropogenic site. Thus, we considered red fox and leopard cat for further analyses and calculated the total detections and RAI in the park and anthropogenic site. We calculated the RAI of red fox and leopard cat for all five sessions and plotted it against the respective locations (latitude and longitude). We used the package *“camtrapR”* in R (v.4.0.5; R Core Team, 2021) to generate the plots. We used the resulting plots to determine the distribution pattern of each mesocarnivore along the habitat gradient covering park and anthropogenic site.

#### Habitat selection

The anthropogenic site was situated at the lower reaches (<2,500 m), while the park was at the higher side (>2,500 m) of the elevation gradient. The park and anthropogenic site demonstrated overlapping values of the covariates of interest like, terrain ruggedness, slope, NDVI and distance to woodland (because of riparian forest at hill base and grasslands at hill brows). For instance, terrain ruggedness overlapped highly (0.3 to 0.6 in anthropogenic site and 0.3 to 0.7 inside park) and elevation had low overlap (1,500 to 2,900 m in anthropogenic site and 2,000 to 4,300 m in park) (Figs. S6 to S10, Table S2). Thus, the habitat characteristics in park and anthropogenic sites were comparable except for the presence of human settlements in the latter. The villages were majorly located near the river at lower reaches on hill slopes, hill base, less rugged terrain, and woodlands. In this condition, species with specific habitat preferences in their natural state might likely differ when exposed to anthropogenically modified habitats (Schuette et al., 2013).

We selected habitat variables based on red fox and leopard cat ecology. We identified five habitat variables as potentially significant predictors where red fox (McDonald et al., 2017; Sacks, Statham & Wittmer, 2017; O’Malley et al., 2018; Martin-Garcia et al., 2022) and leopard cat (Bashir et al., 2014; McCarthy et al., 2015; Can et al., 2020) were likely to occur: elevation, slope, terrain ruggedness, NDVI and distance to woodland (riparian forests at hill base). Red fox preferred alpine meadows (high elevation, moderate slope), rugged terrain, and open and rocky areas (low NDVI) (Weber & Meia, 1996; Murdoch et al., 2016; Kumar, Magar & Kumar Dhamala, 2019; Naseer et al., 2020), and grasslands at hill brow (away from woodland at hill base) (Sacks, Statham & Wittmer, 2017; Rodríguez et al., 2020) in natural forests and used variety of habitats in anthropogenic areas (Mueller, Drake & Allen, 2018; Jahren et al., 2020; Jackowiak et al., 2021). Hence, we expected red fox to show habitat selection towards high rugged, high elevation, moderate slope, low NDVI and away from woodlands inside the park and no habitat selection pattern in anthropogenic site. On the other hand, leopard cat occurred in temperate to sub-alpine forests (>3,000 m, moderate slope) (Mishra, Madhusudan & Datta, 2006; Thapa et al., 2013; Irawan et al., 2020). Leopard cats prefer rugged terrain, tree-covered (high NDVI), and woodland habitats (Ghimirey & Ghimire, 2010; Bashir et al., 2014; Buzzard, Li & Bleisch, 2018; Pin et al., 2022) in natural forests. It used lower reaches and a wide range of habitats in anthropogenic sites (Lorica & Heaney, 2013; Petersen et al., 2019; Wu et al., 2020). Likewise, for leopard cats, we expected to see habitat selection towards high rugged, high elevation, moderate slope, high NDVI, closer to woodlands inside the park and no such pattern in the anthropogenic site.

### Data preparation

We categorised the area surveyed for carnivore faecal samples inside the national park as park (faecal site). These were the areas where we could not conduct camera trapping due to rugged terrain. We used five explanatory variables as the habitat covariates in the park (faecal site): elevation, terrain ruggedness index (TRI), slope, NDVI and distance to woodland (Table S2). We extracted the habitat covariates from the midpoint of each of the 0.5 km trails.

We categorised the camera trapped area inside the national park as park (camera site). Explanatory variables used as habitat covariates in the park (camera site) and anthropogenic site were the same as park (faecal site). We extracted the values of all habitat covariates from each camera trap point location (Table S2).

We checked for the collinearity between habitat covariates in park (faecal site), park (camera site) and anthropogenic site using r values (−1 to 1) as it might reduce the precision of the estimated coefficients (Dormann et al., 2013). None of the covariates was co-related in either the anthropogenic site or park (Figs. S11–S13). The test was performed on the R platform using package *lattice* (Sarkar, 2008).

### Data visualisation

In park (faecal site), we assessed the total number of genetically confirmed faecal samples of red fox and leopard cat. We considered each mesocarnivores’ relative abundance index (RAI) as the number of confirmed faecal samples from each trail (0.5 km). In park (camera site), we calculated the RAI of red fox and leopard cat as the capture rate per 100 trap nights for each camera trap location. Likewise, we calculated the RAI of red fox and leopard cat for each camera trap location using the capture rate per 100 trap nights in the anthropogenic site. We plotted the RAI of the mesocarnivores against the respective habitat covariates for all five sampling sessions in the park (faecal site), park (camera site) and anthropogenic site to account for linear, non-linear patterns and outliers. Most of the RAI vs covariate relationships were non-linear (Figs. S14 to S19); therefore, we chose hierarchical generalized additive modelling (HGAM) (Zuur, 2012; Pedersen et al., 2019) to model each response of mesocarnivores to habitat covariates.

### Statistical analysis

We separately modelled the detections from genetically confirmed mesocarnivore faecal and camera trap data. We performed GAM using “*mgcv”* package (Wood, 2011) in R (v.4.0.5) to assess the habitat selection of mesocarnivores in park (faecal site). Here, we considered the number of genetically confirmed red fox faecal samples (F = 83) from each trail as the response variable. Due to the low sample size of genetically confirmed leopard cat faecal samples (F = 40), we dropped leopard cat for habitat selection analysis. We found a linear relationship between red fox faecal count and elevation (Fig. S17) while data visualisation; hence we did not apply a smoother function. The equation of spline regression (Zuur & Ieno, 2018) used for the additive model for red fox in park (faecal site) was:

(1) $$\eqalign{
  & {{\rm{F}}_{\rm{j}}} = \;\alpha  + f({\rm{elevatio}}{{\rm{n}}_{\rm{j}}}) + f({\rm{TR}}{{\rm{I}}_{\rm{j}}}) + f({\rm{slop}}{{\rm{e}}_{\rm{j}}}) + f({\rm{NDV}}{{\rm{I}}_{\rm{j}}})  \cr 
  & \quad \quad  + f({\rm{distance}}\;{\rm{to}}\;{\rm{woodlan}}{{\rm{d}}_{\rm{j}}}) + {\varepsilon _j} \cr} $$

where ${\rm F_j}$ = number of genetically confirmed red fox faecal samples from each trail, j = each survey trail, α = intercept, *f* = smoother function, Ɛ = residuals. The underlying idea of spline regression is to separate the covariate into k segments (knots) and apply a bivariate linear regression model to the data of each segment. A smoother was obtained by connecting the regression lines for all segments (Zuur & Ieno, 2018). To allow for smooth connections at the knots, we used a cubic regression spline for each covariate (Zuur, 2012). For example, the equation of smoother function for elevation in Eq. (1) becomes:

(2) $$\eqalign{
  & f({\rm{elevatio}}{{\rm{n}}_{\rm{j}}}) = \;{\beta _1} \times {\rm{elevatio}}{{\rm{n}}_{\rm{j}}} + {\beta _2} \times {\rm{elevation}}_{\rm{j}}^{\rm{2}} + {\beta _3} \times {\rm{elevation}}_{\rm{j}}^{\rm{3}}  \cr 
  & \quad \quad \quad \quad \quad \quad  + \sum\nolimits_{p = 1}^{\rm{k}}  \times  ({\rm{elevatio}}{{\rm{n}}_{{\rm{jp}}}}{\rm{ - }}{{\rm{k}}_{\rm{p}}})_ + ^{^3} + {\varepsilon _j} \cr} $$

where j = each survey trail, β = unknown regression parameters, k = number of knots, p = knot positions in the x-axis. We first modelled Gaussian, Poisson and negative binomial distributions without k-value (to avoid the unnecessarily large number of candidate models) to select the best distribution. We then chose the model with the lowest AIC and tested it with possible 8 k-values (2 to 9). Therefore, the number of candidate models for red fox in park (faecal site) was 40: 5 variables × 8 k-values. In park (camera site) and anthropogenic site, we performed HGAM. We used the number of mesocarnivore detections (C) from each camera traps as the response variable and camera operational days as an offset. The park (camera site) and anthropogenic site were used as two zones and incorporated into the model as factors. The equation of the additive model using park (camera site) and anthropogenic sites as factors was:

(3) $$\eqalign{
  & {{\rm{C}}_{\rm{i}}} = \;\alpha  + f({\rm{elevatio}}{{\rm{n}}_{\rm{i}}},\;{\rm{by = fzone}}){\rm{ + }}f({\rm{TR}}{{\rm{I}}_{\rm{i}}},\;{\rm{by = fzone}}){\rm{ + }}f({\rm{slop}}{{\rm{e}}_{\rm{i}}},\;{\rm{by = fzone}})  \cr 
  & \quad \quad  + f({\rm{NDV}}{{\rm{I}}_{\rm{i}}},\;{\rm{by = fzone}}) + f({\rm{distance}}\;{\rm{to}}\;{\rm{woodlan}}{{\rm{d}}_{\rm{i}}},\;{\rm{by = fzone}})  \cr 
  & \quad \quad  + {\rm{offset}}\;({\rm{logdays}}){\rm{ + }}{\varepsilon _{\rm{i}}} \cr} $$

where C_i_ = number of mesocarnivore detections from each camera trap location, i = each camera trap location, α = intercept, *f* = smoother function, logdays = logarithm of camera operational days, fzone = park (camera site) and disturbed site, Ɛ = residuals. The number of candidate models for each mesocarnivore in park (camera site) and anthropogenic site was 40: (5 variables × 8 k-values). We plotted the response curves (detections) of mesocarnivores against each significant explanatory variable for park (faecal site), park (camera site) and anthropogenic site, respectively. We used the package “*ggplot2”* (Wickham, 2016) and *“ggeffects”* (Lüdecke, 2018) in R (v.4.0.5) for plotting the response curves. We evaluated the significance of the explanatory variables in each model using the p-values from the Wald statistics in the *“mgcv”* package. We also evaluated the effective degrees of freedom (edf) for each covariate to understand the scale of non-linearity captured by the model. The shaded area in the resulting plots represents 95% point-wise confidence bands of the smoother covariates (Zuur, 2012).

### Model selection

We selected the best model based on the lower Akaike Information Criterion (AIC), overdispersion values (OD) (Zuur, 2012; Zuur, Hilbe & Ieno, 2013) and k values with the best ecological meaning (Figs. S14 to S19). For park (faecal site), we selected negative binomial GAM as the best distribution for the habitat selection model for red fox. We chose the best model out of 40 candidate models with AIC = 241.4 and OD = 1.2. For the combined model of park (camera site) and anthropogenic site, we selected negative binomial distribution as the best distribution. We chose the best model each for red fox (AIC = 744.8, OD = 2.8) and leopard cat (AIC = 988.9, OD = 2.2) out of 40 candidate models.

### Model validation

We performed concurvity test to check for non-linear dependencies in the predictor variables for each of the best models in park (faecal site) and combined model of park (camera site) and anthropogenic site (Amodio, Aria & Ambrosio, 2014). We did not find any concurvity in the predictor variables (Figs. S20–S22). We conducted homogeneity test to check for any pattern in the residuals due to model misspecification (Pearson residuals *vs* fitted values) (Zuur, Leno & Smith, 2007; Zuur, 2012; Zuur, Hilbe & Ieno, 2013). We performed independence test to check for patterns in residuals due to any covariate (Pearson residual *vs* covariates) (Zuur, 2012; Zuur & Ieno, 2018). We did not find any clear pattern in either of the Pearson residual vs fitted values plots (indicating homogeneity, Figs. S23, S24) or Pearson residual *vs* covariate plots (showing independence, Figs. S25, S26) for red fox and leopard cat in park (faecal site), park (camera site) and anthropogenic site. We checked for spatial dependency using semi-variogram plots (residual *vs* space) using package *“gstat”* (Pebesma, 2004) in R (v.4.0.5). The semi-variogram plots (Pearson residual *vs* space) indicated no spatial dependency in the photo captures (C) of red fox and leopard cat to the distance between sampling locations in park (faecal site), park (camera site) and anthropogenic site (Figs. S27, S28). We also investigated the influential observations in the model using cook’s distance and found four influential observations for red fox in the anthropogenic site and were not dropped (Figs. S29, S30).

#### Non-metric multidimensional scaling for factors responsible for space usage by mesocarnivores in anthropogenic site

After assessing the habitat selection by mesocarnivores in the anthropogenic site, we investigated the driving factors that might be possibly responsible for space usage in the anthropogenic site. Since the villages were small and located at a distance, the type of human attributes changed on moving outwards from the village core. For instance, the village centre had the maximum number of houses surrounded by a few agricultural plots. In contrast, the village edges had fewer houses, mainly farming fields and human trails. Therefore, we categorised the camera trap locations into three human attributes: houses, agricultural plots, human trails, and three topography types: hill base, hill slope and hill top. Human disturbances like livestock, humans and dogs also varied with increasing distance to the village. To depict the variation of the three human attributes, three village topography types and three human disturbance variables, we divided the distance to village into three classes; “within village” (0 to 300 m), “near village” (300 to 600 m) and “away from village” (>600 m) based on distances between camera trap locations and village centroids (average village area radius 300 m). We calculated the RAI (capture rate per 100 trap nights) of red fox, leopard cat, human, dog and livestock for all the categories under three distance classes. We used Non-metric Multidimensional Scaling (NMDS) (Clarke, 1993) to understand the factors responsible for space usage of mesocarnivores in villages. NMDS was performed in R using “*vegan”* package (Oksanen et al., 2020). We modelled the ordination over different dimensions (1, 2 and 3) with Bray-Curtis distance and selected the best model based on the lowest stress value (less than 0.05 is a good fit) (Zuur, Leno & Smith, 2007).

## Results

### Distribution pattern

**Figure 2:**
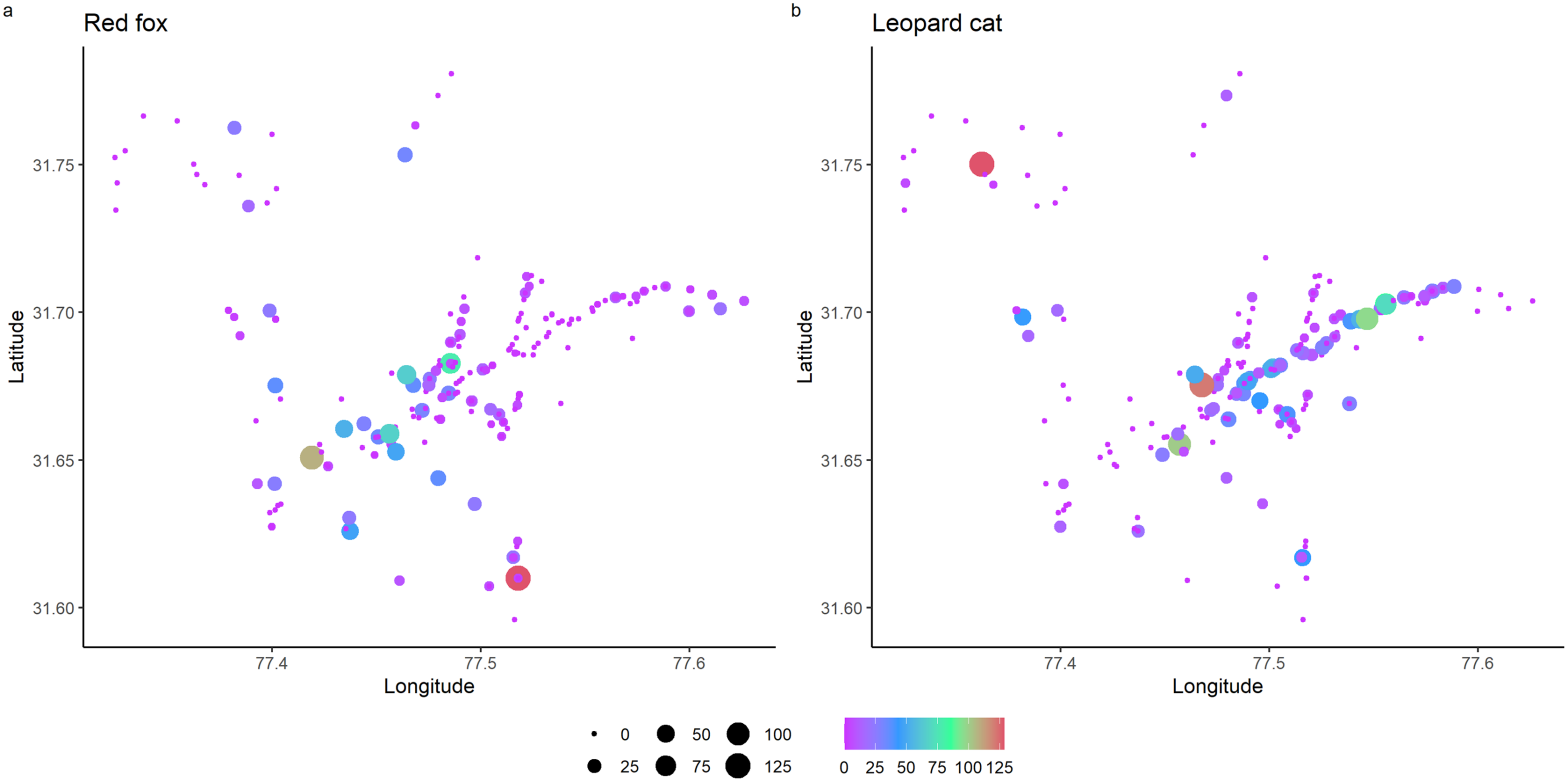
Relative abundance index (capture rate per 100 trap night) of mesocarnivores in GHNPCA during the sampling period from 2017 to 2019.

Total detections of red fox and leopard cat were 344 and 524, respectively. In park, red fox and leopard cat detections were 119 and 405, and in the anthropogenic site, the detections were 214 and 130, respectively. RAI of red fox in the park and anthropogenic site were 1.54 (0.14) and 8.77 (0.39), and of leopard cat were 4.47 (0.29) and 5.01 (0.28), respectively (values in the parenthesis depict standard error). Figure 2 shows the RAI of red fox and leopard cat along the habitat gradient, including all five camera trapping sessions.

### Habitat selection

**Figure 3:**
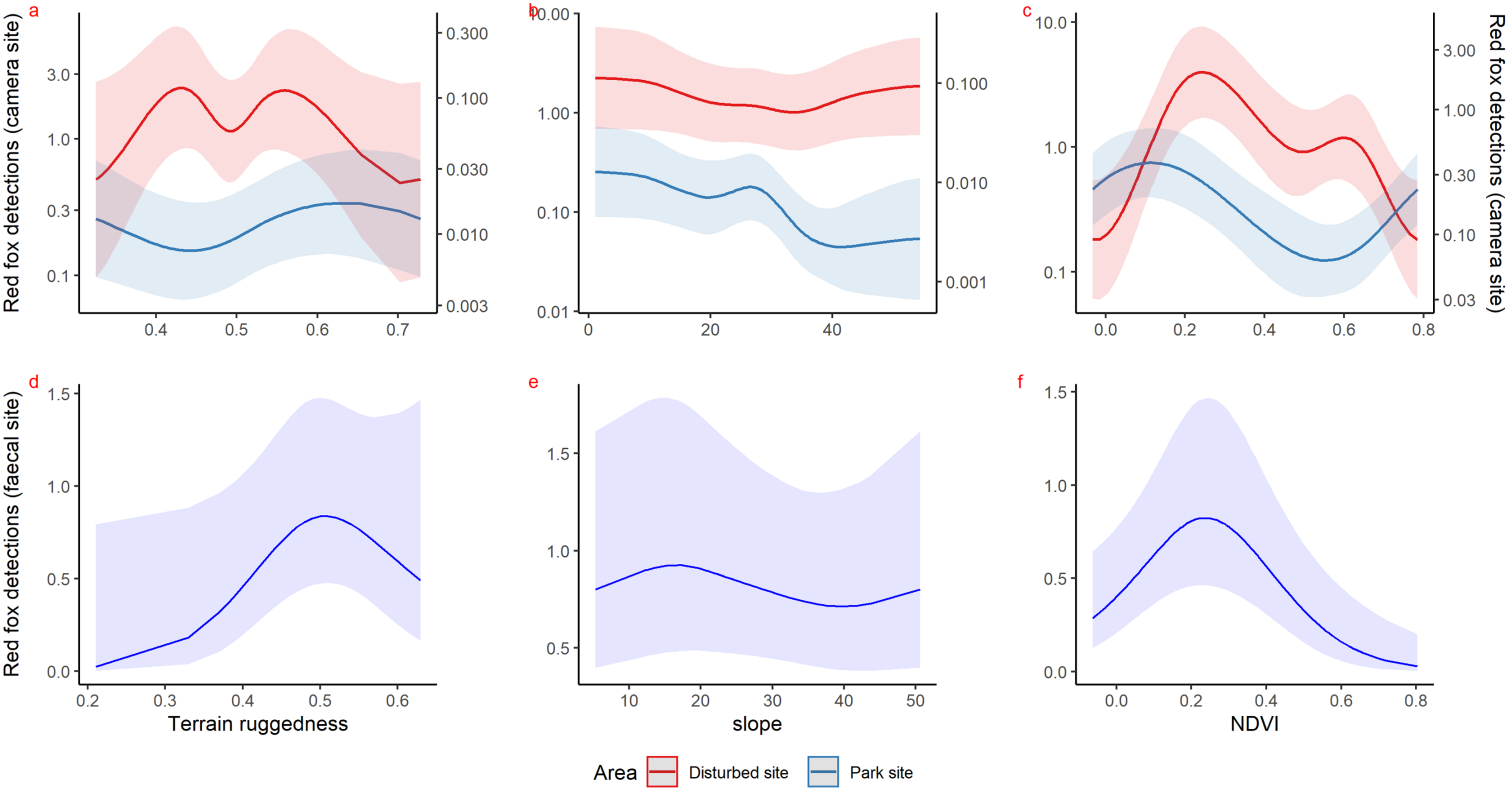
Response curves of the best models from HGAM (negative binomial distribution) showing habitat selection of red fox in park (camera site, blue line) and anthropogenic site (red line) (A to C) and park (faecal site, blue line) (D to F).

Inside park, GAM results of red fox in remote high elevation areas using genetically confirmed faecal samples revealed preference for certain habitats. Red fox occurred mostly at high rugged terrain (0.5 TRI, *p*-value: 0.0009, edf = 1.8) (Fig. 3D), low slope (edf = 0.7) (Fig. 3E) and low NDVI (0.2, *p*-value: 0.0002, edf = 1.9) (Fig. 3F) sites.

**Figure 4:**
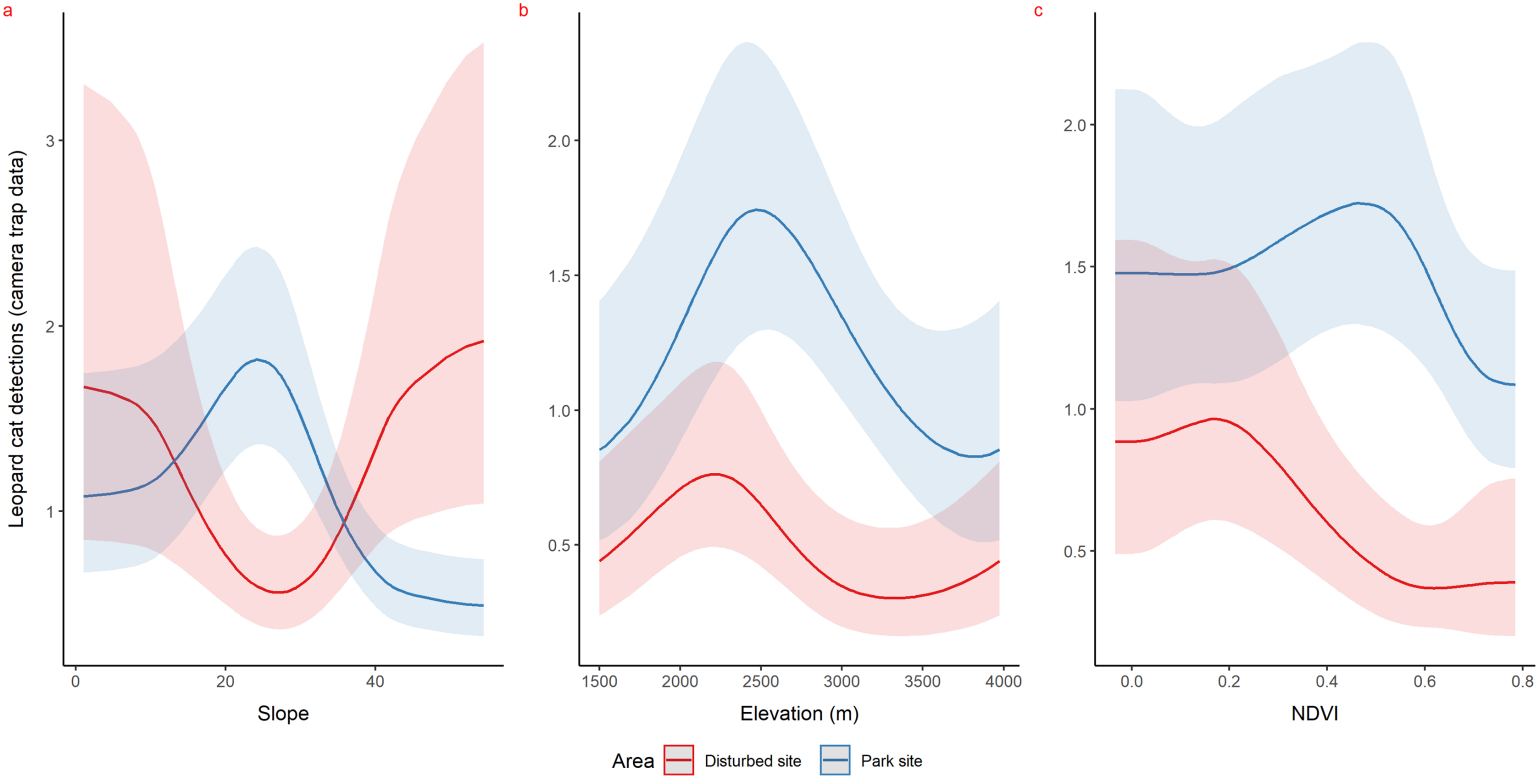
Response curves of the best models from HGAM (negative binomial distribution) showing habitat selection of leopard cat in park (camera site, blue line) and anthropogenic site (red line) (A to C). Due to low faecal sample size leopard cat was dropped for park faecal site.

Using camera traps, we found similar habitat preferences of red fox inside the park. Red fox preferred areas with rugged terrain (0.6 TRI, *p*-value: 0.0009, edf = 1.4) (Fig. 3A), moderate slope (20°, *p*-value: 0.0004, edf = 2.8) (Fig. 3B) and low NDVI (0.1, *p*-value: 0.0002, edf = 2.2) (Fig. 3C). Also, cameras detected leopard cat most commonly at moderate slope (20°, *p*-value: 0.0001) (Fig. 4A) and high NDVI (0.4 to 0.6, *p*-value: 0.03) (Fig. 4C) inside park. Although leopard cat occurrence decreased with increasing elevation, we found a peak in the smoother curve at 3,000 m elevation (Fig. 4B) (*p*-value: 0.0005). All the *p*-values mentioned above refer to the significance of the smoother covariate, not the predicted mesocarnivore counts at a particular value of the smoother covariate. On the other hand, neither red fox nor leopard cat showed any habitat-specific selection in the anthropogenic site. As we did not find any prominent peak in the smoother curves of red fox and leopard cat with either of the habitat covariates (Figs. 3A–3C and 4A–4C). Except for an additional peak at 0.6 NDVI (edf = 2.9) in case of red fox (Fig. 3C). The other smoother curves of habitat covariates in relation to leopard cat and red fox habitat selection were provided in Figs. S31 and S32, respectively. Although the GAM models revealed species-specific habitat selection but most of the non-linear effect of habitat covariates on red fox and leopard cat detections were small (indicated by small edf values) due to low detections at camera trap locations. Hence precautions should be taken while interpreting results considering ecological meaning.

### Factors responsible for space usage by mesocarnivore in anthropogenic site

**Figure 5:**
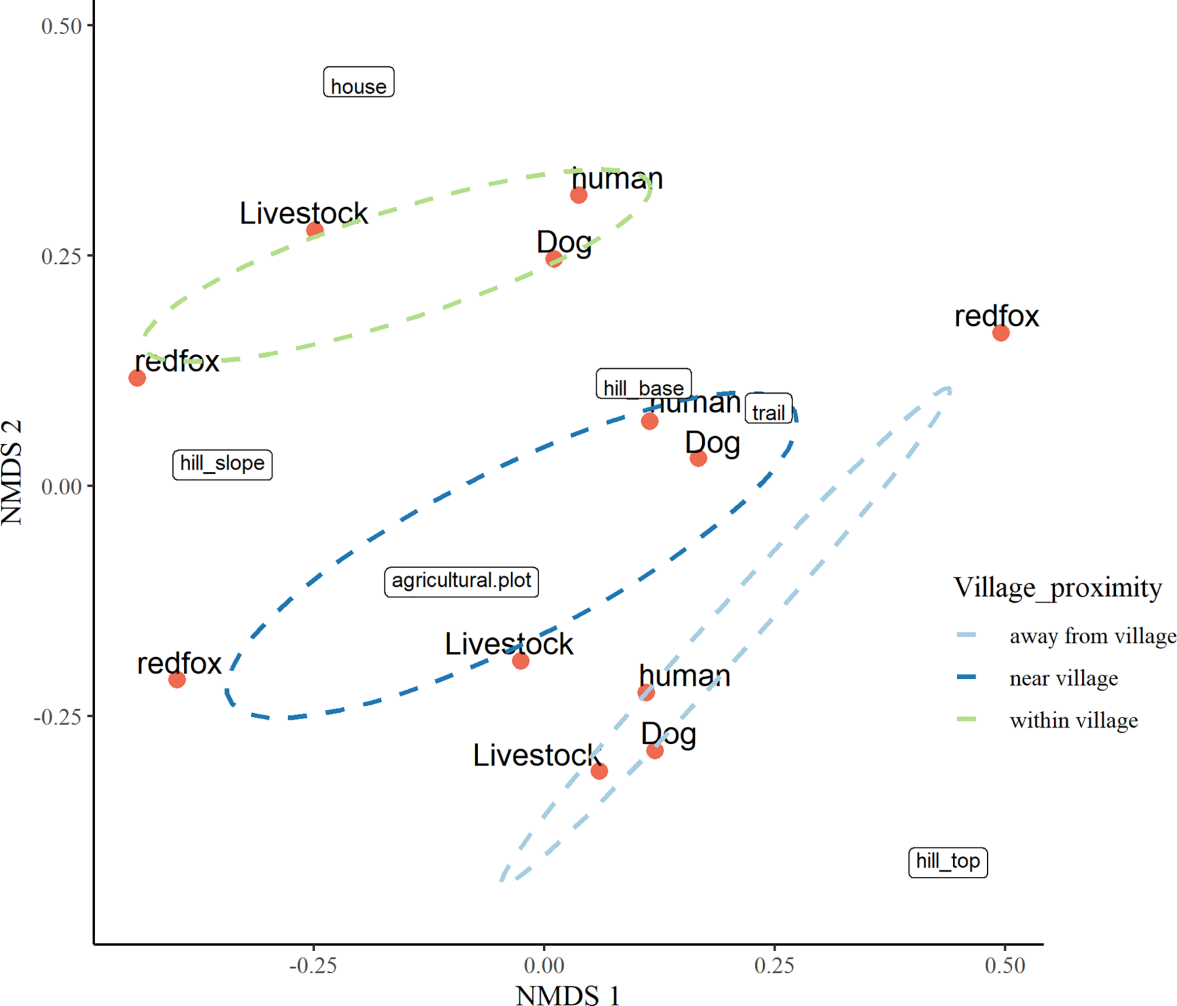
NMDS ordination plot showing factors responsible for space use by red fox in anthropogenic site (ecozone).

**Figure 6:**
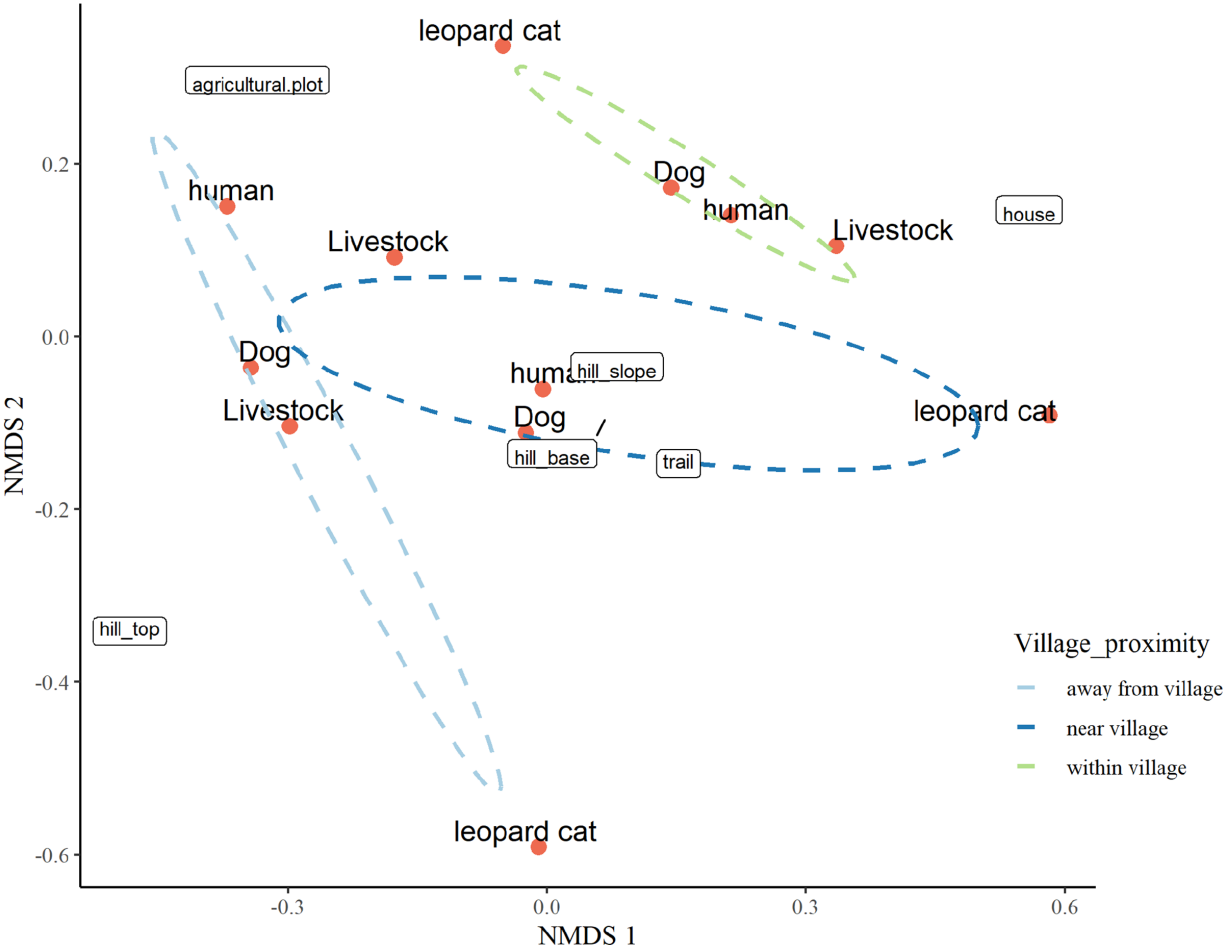
NMDS ordination plot showing factors responsible for space use by leopard cat in anthropogenic site (ecozone).

The three-dimensional NMDS ordination identified the factors responsible for intensive site usage in villages by red fox and leopard cat (stress value 0.017; Figs. 5, 6; Tables S3, S4). We have provided the shepherd diagrams (a graphical representation of the stress values) of all the tested models for red fox and leopard cat in Figs. S30 and S31. We found the relationship of red fox and leopard cat to three human attributes (house, agricultural plot, human trail), three village topography types (hill base, hill slope, hill top) and three human disturbance variables (human, dog, livestock). Within villages, red foxes used sites near households and hill slopes, showing positive relation to livestock (Fig. 5). Red foxes near the village used more agricultural plots and hill slopes, showing positive relation to livestock. Away from the village, human-made village trails and hill base were the sites where red foxes mostly occurred. Red fox showed negative relation to the disturbance variables; human and dog. Leopard cats within villages primarily used agricultural plots and hill slopes (Fig. 6). Near the village, they mostly used houses and hill slopes (Fig. 6). Away from the village, leopard cats used human trails and hill base. Overall, leopard cats showed negative relation to humans, dogs and livestock.

## Discussion

### Distribution in human-modified – natural gradient

The study was the first attempt to understand the effect of fine-scale habitat modification using habitat covariates like slope, elevation, ruggedness and NDVI on the distribution of mesocarnivores using complementary (camera traps and molecular) approaches in GHNPCA in the western Himalaya. We observed variation in the site intensity usage of mesocarnivore in the anthropogenic site-park gradient, where the anthropogenic site is located at lower reaches and park at higher elevations. For instance, the overall capture rate (RAI) of red fox and leopard cat in the anthropogenic site was higher than in the park. The result contradicts the established literature from GHNPCA in 1999, where red fox and leopard cat were recorded only inside the park at high elevations (Vinod & Sathyakumar, 1999). The shift in presence of mesocarnivores from less disturbed forested habitats to human dominated habitats in the last two decades affirms the increasing human population and its associated land usages in the landscape and its effect on native wildlife. In the previous research, some results were comparable due to the substantial walk effort of 290 km in anthropogenic site and 867 km in the park. However, carnivore sign surveys on human trails were not as robust compared to camera trapping used in this study. In the current study, the higher RAI of red fox and leopard cat in the anthropogenic site than in park explains the intensive site usage by the native wildlife in the human-dominated areas in GHNPCA. The result was analogous to studies by Lorica & Heaney (2013) and Reshamwala et al. (2018), where red fox and leopard cat occurred more frequently in anthropogenically disturbed areas like human settlements, agricultural plots, *etc*. The possible reason can be that the disturbed areas favour the native wildlife for readily available food resources like carrions, rodents, kitchen wastes, *etc*. And as a result, they use these areas more frequently than less disturbed habitats. The variation in site usages along the disturbed to natural gradient, elucidates the role of mesocarnivores as indicators of habitat quality (Goad et al., 2014; Šálek, Drahníková & Tkadlec, 2015; Wang, Allen & Wilmers, 2015). The differences in intensive site usage by native wildlife point towards habitat modifications in terms of increasing households, expanding agricultural plots, availability of human-induced food resources, *etc*. at the lower reaches of GHNPCA. After assessing the overall distribution pattern, we further investigated the site-specific occurrences of red fox and leopard cat using 3^rd^ order habitat variables in park and anthropogenic site.

### Species-specific habitat selection

Red fox and leopard cat showed habitat selection in park that differed from anthropogenic site. Inside the park, red fox mainly occurred in high rugged (>0.5), moderate slopes and open and rocky (low NDVI; 0.1 to 0.2) locations depicting temperate grasslands, sub-alpine scrubs and alpine meadows (Rossi et al., 2019). The result aligned with the previous study by Vinod & Sathyakumar (1999) in GHNPCA and other established literature, Halpin & Bissonette (1988) and Cavallini & Lovari (1991). The result explains that given less human disturbance, the red foxes tend to occupy the habitat according to their natural behaviour and ecology. However, in the anthropogenic site, red foxes used a variety of habitats. They did not show any habitat-specific selection, unlike in the park, indicating red foxes using sites which were out of their natural behaviour in disturbed habitats. The change in the occurrence of red fox in selected habitats inside park to a wide range of habitats in anthropogenic site revealed its flexibility in humanized environments (Díaz-Ruiz et al., 2016) and exploitation of niches that form in the wake of human activities (Jahren et al., 2020). Furthermore, leopard cat mainly occurred in moderate slope, high elevation and high NDVI inside park, which was upper temperate forests with dense tree-cover. The result was supported by the aforementioned study in GHNPCA in 1999 and Bashir et al. (2014) in eastern Himalaya, suggesting leopard cats prefer sites as per their ecology in the natural habitats in GHNPCA. Interestingly we did not find such habitat selection in the anthropogenic site, and leopard cats used a variety of locations irrespective of habitat preferences, including open areas (low NDVI). The result was analogous to Rajaratnam et al. (2007) and Izawa et al. (2009), where leopard cat utilised a wide range of habitats in a human-modified landscape. We could perceive that leopard cats changed their site selection out of their natural behaviour, based on levels of anthropogenic disturbances in different habitats. In general, leopard cats inhabit diverse habitat types, but they respond differently to the extent of anthropogenic exposures. For instance, in Bashir et al. (2014), leopard cats were reported to have a low tolerance to deviations from their preferred habitat in the eastern Himalaya. Still, it manages to thrive in varied landscapes. Overall, red fox and leopard cat in GHNPCA showed differences in habitat selection in park and anthropogenic sites, reflecting species-specific sensitivities to habitat changes (Recio et al., 2015; Riggio et al., 2018). The difference in habitat selection revealed their opportunistic and human adapter behaviour (Ditchkoff, Saalfeld & Gibson, 2006) that is an outcome of resource utilisation (Zhao et al., 2020), especially in resource-scarce and rugged landscapes in the western Himalaya. The change in selection of specific habitats inside the park to a wide range of habitats in anthropogenic site by red fox and leopard cat revealed impact of human disturbances on a finer scale (Lorica & Heaney, 2013). This argument can be further supported by the fact that human habitations in GHNPCA were majorly located in the anthropogenic site. Thereby resulting into adaptive behaviour of mesocarnivores through shift in habitat choices for utilising resources in the anthropogenic habitats (Duduś et al., 2014). The opportunistic behavior of mesocarnivores was further supported by the site usages close to different human attributes in the anthropogenic sites.

### Factors responsible for space usage around human habitation

The NMDS ordination plots revealed that both the mesocarnivores showed close association with human attributes like houses, agricultural plots and human trails in villages. The choices of these attributes varied with increasing distance from villages reflecting resource utilisation at different scales. For instance, space usage by red foxes close to households inside villages, agricultural plots in the village vicinity, and trails outside villages relate to utilising anthropogenic food subsidies in villages. The findings were similar to Ghoshal (2011) and Ghoshal et al. (2016), where red foxes used sites close to villages and agricultural plots for anthropogenic food subsidies. It indicates that houses and agricultural plots in anthropogenic site of GHNPCA are the possible sources of food and hence responsible for intensive site usage by red foxes in these areas. Furthermore, red fox was positively related to livestock like goats and sheep, suggesting the possibility of livestock depredation, which aligned with Aryal, Sathyakumar & Kreigenhofer (2010) and Maheshwari & Sathyakumar (2020). Also, red foxes showed a negative relationship with humans and dogs in villages possibly due to disturbance and threat from the competitive carnivore (Gil-Fernández et al., 2020; Reshamwala et al., 2021). Interestingly hill slopes were the most frequently used sites within and near villages. This result was complementary to Reshamwala et al. (2021), where red foxes used the hill slopes for denning sites with minimal human disturbance. Unlike red fox, leopard cat showed close association with agricultural plots within villages and houses near villages which was in accordance with the study by Rajaratnam et al. (2007) and Vitekere et al. (2020). Knowing the importance of rodents in leopard cat diet, the abundance of rodents in these areas explains the space usage near agricultural fields and houses (Lorica & Heaney, 2013). The negative relation of leopard cats to human presence and dogs revealed their sensitivity towards disturbance and inter-specific avoidance, which aligned with Oh et al. (2010), Cheyne & MacDonald (2011) and Weng et al. (2022). Overall, for red foxes presence of houses played a crucial role in intensive space usage, whereas for leopard cats, agricultural plots were the important factors for frequent space usage in the anthropogenic site of GHNPCA. Hence our results suggest that human-induced disturbances like houses and agricultural plots are the potential factors responsible for thorough space usage by the native wildlife in the study area.

The distribution and habitat selection of mesocarnivores along the human-modified and natural gradient clearly showed the impact of increasing and expanding anthropogenic activities around GHNPCA. The outcome was similar to Schuette et al. (2013) and Ditchkoff, Saalfeld & Gibson (2006), where mesocarnivores showed signs of adaptation to expanding human habitations, indicating mesocarnivore adaptations to human-modified habitats in GHNPCA. The ecozone around the park was delineated as a buffer area to lower anthropogenic activity’s direct pressure on the GHNPCA boundary. Although land settlements and agricultural expansions occurred earlier (Tucker, 1997), the current status of human habitation around GHNPCA needs re-evaluation to implement effective conservation practices. One of the caveats of human residences adjacent to natural forests adds to the availability of anthropogenic food sources like garbage dumps, agricultural products, kitchen wastes and livestock carrions in village areas (Randa & Yunger, 2006; Newsome et al., 2015). Mesocarnivores, an opportunistic feeder, roam around these areas for food subsidies (Reshamwala et al., 2018). Eventually, these shared spaces can be the most probable zones for zoonotic disease transmissions and human-wildlife conflict (Namusisi et al., 2021). In particular, the disease spread can be bi-directional, *i.e*., from red fox, leopard cat to domestic animals or humans (Plumer et al., 2014; Chhabra & Muraleedharan, 2016; Nadin-Davis et al., 2021) or humans, livestock to the mesocarnivores (Clark et al., 2018; Ng et al., 2019). Concurrently, the availability of livestock and crop (like maize) close to GHNPCA also confers the exposure of native large carnivores like leopard and Himalayan black bear to more vulnerable habitat conditions and conflict probabilities (Sathyakumar, 2000; Chauhan, 2003; Charoo, Sharma & Sathyakumar, 2011; Naha et al., 2020a). Past evidence and our results suggest that wildlife in rural areas do not exhibit the same habitat preferences as their natural counterparts because of adaptation to human-induced modifications (Ditchkoff, Saalfeld & Gibson, 2006). Managers in such situations face challenges in addressing problems associated with rural wildlife and expanding human habitation. There is a need for management strategies for human habitation expansion and proper garbage disposal practices, primarily in the anthropogenic site. In this context, we posit the need for mitigation efforts aimed at expansion of human habitation and systematic garbage disposal practices at the human-wildlife interface to safeguard future disease outbreaks and conflict risks that address sustainable development goals.

### Limitations

Our study had few limitations due to inherent challenges. The study area was devoid of any paved road. Hence, the entire sampling from 2017 to 2019 was carried out on foot. The inaccessible terrain enabled us to conduct either camera trapping or carnivore faecal sampling beyond 4,300 m elevation inside the national park. It restricted us from broadening our understanding of distribution and habitat selection of mesocarnivores at such high altitudes. Also, due to logistical constraints, the number of camera traps in the anthropogenic site was less than in the park, although the capture rates and detections of mesocarnivores in anthropogenic site were comparable to that of the park implying representative area coverage by camera trapping in anthropogenic site. Due to harsh environmental conditions, no fieldwork was conducted in the monsoon (August–September) and snow (January–March) seasons. Therefore, this study was carried out only in the accessible months from April to July and October to December. We excluded data derived from cameras not functioning correctly (due to camera failure, battery failure and heavy snowfall), resulting in the non-detection of the target species. Also, camera placements, orientation, temperature differences, faecal sample detection and degradation due to logistic limitations for storage added to species non-detection. To overcome the non-detection issue, we conducted camera trapping and faecal sample collection in five sessions by walking more than 400 km to sample each trail and maximise species detections. The detections were entirely satisfactory, as revealed by the GAM results. Our results depicted changes in habitat selection in modified habitats and factors responsible for space usage in villages using camera trapping and faecal sampling approach. However, we recommend careful insights while placing camera traps (especially at high altitudes) and collecting faeces in future analogous studies.

## Conclusion

As development continues, it is crucial to understand how carnivores might respond to increased human expansions and the factors that might put carnivores and humans at increased risk of conflict and disease spread. Mesocarnivore distribution and habitat selection along the anthropogenic site-park gradient in GHNPCA clearly showed the influence of anthropogenically modified habitat. The effects can harm other large carnivores like leopard and Himalayan black bear, leading to negative interactions like in other parts of western Himalaya. The anthropogenic site is an interface area where human habitations extend toward the natural habitat. Concurrently mesocarnivores from adjacent forest areas utilise the human habitations. Therefore, it is crucial to reinforce the conservation practices in anthropogenic site to control the habitat modifications adjacent to natural habitats and reduce the anthropogenic effects on native wildlife. It took more than 30 years (1980 to 2014) from inception (Pandey & Wells, 1997) to realization of GHNPCA as a World Heritage Site (UNESCO, 2014: https://whc.unesco.org/en/list/1406/), but the current situation in the buffer zone of GHNPCA threatens its protection status in future. There is a need to implement mitigation strategies in the human-wildlife interface areas to regulate human habitation expansions and its associated caveats to balance the spheres of humans and wildlife in the study area.

## Supplemental Information

10.7717/peerj.13993/supp-1 Supplemental Information 1 Map of GHNPCA showing sampling locations in Session 1: park (control) site 2 (camera trap locations = 28) and anthropogenic site (camera trap locations = 31) during April to July 2017.

10.7717/peerj.13993/supp-2 Supplemental Information 2 Map of GHNPCA showing sampling locations in Session 2: park site 1 (faecal sample survey trails = 45), park site 2 (camera trap locations = 47) and anthropogenic site (camera trap locations = 31) during October to December 2017. (park = control).

10.7717/peerj.13993/supp-3 Supplemental Information 3 Map of GHNPCA showing sampling locations in Session 3: park site 1 (faecal sample survey trails = 11), park site 2 (camera trap locations = 35) and anthropogenic site (camera trap locations = 5) during April to July 2018. (park = control).

10.7717/peerj.13993/supp-4 Supplemental Information 4 Map of GHNPCA showing sampling locations in Session 4: park site 1 (faecal sample survey trails = 24), park site 2 (camera trap locations = 52) and anthropogenic site (camera trap location = 30) during October to December 2018. (park = control).

10.7717/peerj.13993/supp-5 Supplemental Information 5 Map of GHNPCA showing sampling locations in Session 5: park site 1 (faecal sample survey trails = 45), park site 2 (camera trap locations = 58) and anthropogenic site (camera trap locations = 23) during April to June 2019. (park = control).

10.7717/peerj.13993/supp-6 Supplemental Information 6 Box whiskers plot showing range of values of terrain ruggedness index in anthropogenic site, park (camera site) and park (faecal site).

10.7717/peerj.13993/supp-7 Supplemental Information 7 Box whiskers plot showing range of values of elevation in anthropogenic site, park (camera site) and park (faecal site).

10.7717/peerj.13993/supp-8 Supplemental Information 8 Box whiskers plot showing range of values of slope in anthropogenic site, park (camera site) and park (faecal site).

10.7717/peerj.13993/supp-9 Supplemental Information 9 Box whiskers plot showing range of values of NDVI in anthropogenic site, park (camera site) and park (faecal site).

10.7717/peerj.13993/supp-10 Supplemental Information 10 Box whiskers plot showing range of values of distance from woodland in anthropogenic site, park (camera site) and park (faecal site).

10.7717/peerj.13993/supp-11 Supplemental Information 11 Collinearity between habitat covariates used in generalised additive modelling for red fox (rf) in park (faecal site).

10.7717/peerj.13993/supp-12 Supplemental Information 12 Collinearity between habitat covariates used in generalized additive modelling for leopard cat (a) and red fox (b) in park (camera site). Leopard cat = lc, Red fox = rf.

10.7717/peerj.13993/supp-13 Supplemental Information 13 Collinearity between habitat covariates used in generalized additive modelling for leopard cat (a) and red fox (b) in anthropogenic site. Leopard cat = lc, Red fox = rf.

10.7717/peerj.13993/supp-14 Supplemental Information 14 Scatter plot showing number of confirmed leopard cat faecal sample against habitat covariates in park (faecal site) in 4 sessions: October–December 2017 (a to e), April–July 2018 (f to j), October–December 2018 (k to o) and April–June 2019 (p to t). No.

10.7717/peerj.13993/supp-15 Supplemental Information 15 Scatter plot showing leopard cat photo capture rate per 100 trap night against habitat covariates in park (camera site) in 5 sessions: April–July 2017 (a to e), October–December 2017 (f to j), April–July 2018 (k to o), October–December 2018 (p to t) and A.

10.7717/peerj.13993/supp-16 Supplemental Information 16 Scatter plot showing leopard cat photo capture rate per 100 trap night against habitat covariates in anthropogenic site in 5 sessions: April–July 2017 (1 to 5), October–December 2017 (6 to 10), April–July 2018 (11 to 15), October–December 2018 (16 to 20).

10.7717/peerj.13993/supp-17 Supplemental Information 17 Scatter plot showing number of confirmed red fox faecal sample per 500m trail against habitat covariates in park (faecal site) in 4 sessions: October–December 2017 (a to e), April–July 2018 (f to j), October–December 2018 (k to o) and April–June 2019 (p t.

10.7717/peerj.13993/supp-18 Supplemental Information 18 Scatter plot showing red fox photo capture rate per 100 trap night against habitat covariates in park (camera site) in 5 sessions: April–July 2017 (a to e), October–December 2017 (f to j), April–July 2018 (k to o), October–December 2018 (p to t) and April.

10.7717/peerj.13993/supp-19 Supplemental Information 19 Scatter plot showing red fox photo capture rate per 100 trap night against habitat covariates in anthropogenic site (ecozone) in 5 sessions: April–July 2017 (1 to 5), October–December 2017 (6 to 10), April–July 2018 (11 to 15), October–December 2018 (16 t.

10.7717/peerj.13993/supp-20 Supplemental Information 20 Concurvity plot showing non-linear independencies in the predictor variables used in GAM for habitat selection modelling of red fox in park (faecal site).

10.7717/peerj.13993/supp-21 Supplemental Information 21 Concurvity plots showing non-linear independencies in the predictor variables used in HGAM for habitat selection modelling of red fox in park (camera site) and anthropogenic site.

10.7717/peerj.13993/supp-22 Supplemental Information 22 Concurvity plots showing non-linear dependencies in the predictor variables used in HGAM for habitat selection modelling of leopard cat in park (camera site) and anthropogenic site.

10.7717/peerj.13993/supp-23 Supplemental Information 23 Graph showing pearson residuals *vs* fitted values of the best models from HGAM (negative binomial distribution) for leopard cat in park (camera site) and anthropogenic site. Due to faecal low sample size, leopard cat was dropped from park (faecal site).

10.7717/peerj.13993/supp-24 Supplemental Information 24 Graph showing pearson residuals *vs* fitted values of the best models from HGAM (negative binomial distribution) for red fox in (a) park (faecal site) (b) park (camera site) and anthropogenic site.

10.7717/peerj.13993/supp-25 Supplemental Information 25 Graph showing pearson residuals *vs* covariates of the best models from HGAM (negative binomial distribution) for leopard cat in park (camera site) and anthropogenic site (a to e). Due to faecal low sample size, leopard cat was dropped from park (faecal si.

10.7717/peerj.13993/supp-26 Supplemental Information 26 Graph showing pearson residuals *vs*. covariates of the best models from HGAM (negative binomial distribution) for red fox in park (faecal site) (a to e), and park (camera site) and anthropogenic site (f to j).

10.7717/peerj.13993/supp-27 Supplemental Information 27 Semi variogram plots showing spatial independencies in the HGAM model (negative binomial) for leopard cat in park (camera site) and anthropogenic site. Due to faecal low sample size, leopard cat was dropped from park (faecal site).

10.7717/peerj.13993/supp-28 Supplemental Information 28 Semi variogram plots showing spatial independencies in the HGAM model (negative binomial) for red fox in (a) park (faecal site) and (b) park (faecal site) and anthropogenic site.

10.7717/peerj.13993/supp-29 Supplemental Information 29 Cook’s distance values for HGAM model of leopard cat in park (camera site) and anthropogenic site. The y-axis shows the Cook’s distance value when a particular observation (x-axis) is dropped.

10.7717/peerj.13993/supp-30 Supplemental Information 30 Cook’s distance values for GAM model for red fox in park (faecal site) (left) and park (camera site) and anthropogenic site (right). The y-axis shows the Cook’s distance value when a particular observation (x-axis) is dropped.

10.7717/peerj.13993/supp-31 Supplemental Information 31 Graphs showing curves of covariates as an output of habitat selection modelling of leopard cat using HGAM.

10.7717/peerj.13993/supp-32 Supplemental Information 32 Graphs showing curves of covariates as an output of habitat selection modelling of red fox using HGAM for park and anthropogenic site.

10.7717/peerj.13993/supp-33 Supplemental Information 33 Shepherd’s diagram from NMDS ordination of leopard cat in ecozone showing increase in number of dimensions (from left to right; k = 1, k = 2, k = 3). With increasing k values the linear fit also increases depicting k = 3 (extreme right) the best fit m.

10.7717/peerj.13993/supp-34 Supplemental Information 34 Shepherd’s diagram from NMDS ordination of red fox in ecozone showing increase in number of dimensions (from left to right; k = 1, k = 2, k = 3). With increasing k values the linear fit also increases depicting k = 3 (extreme right) the best fit model.

10.7717/peerj.13993/supp-35 Supplemental Information 35 Session wise sampling details (camera trapping and faecal sample collection) in GHNPCA during 2017 to 2019. Park site 1 shows number of surveyed trails for carnivore faecal sample collection, Park site 2 and anthropogenic site indicates number of camera trap location

10.7717/peerj.13993/supp-36 Supplemental Information 36 Details of explanatory variables used in generalised additive modelling.

10.7717/peerj.13993/supp-37 Supplemental Information 37 RAI (capture rate per 100 trap night) of red fox and human disturbance variables (human, dog, livestock) in ecozone in GHNPCA used for NMDS. Village proximity refers to distance of camera trap locations from village centroids (within; 0–300 m, near; 300–600 m, away; >600 m).

10.7717/peerj.13993/supp-38 Supplemental Information 38 RAI (capture rate per 100 trap night) of leopard cat and human disturbance variables (human, dog, livestock) in ecozone in GHNPCA used in NMDS. Village proximity refers to distance of camera trap locations from village centroids (within; 0–300 m, near; 300–600 m, away; >600 m).

10.7717/peerj.13993/supp-39 Supplemental Information 39 Red fox and leopard cat confirmed faecal sample counts from each surved trails (trail ID) and habitat covariates extracted from the mid point of each trail in national park (control site 1).

10.7717/peerj.13993/supp-40 Supplemental Information 40 Red fox and leopard cat detections and habitat covariates from each camera trap point locations (station ID) in national park (control site 2).

10.7717/peerj.13993/supp-41 Supplemental Information 41 Red fox and leopard cat detections and habitat covariates from each camera trap point locations (station ID) in ecozone (test site).

## Additional Information and Declarations

### Data Availability

The following information was supplied regarding data availability: The raw data is available in the Supplemental Files.
