# Supplementary figures and images for: Understanding the distribution and fine-scale habitat selection of mesocarnivores along a habitat quality gradient in western Himalaya
Source: PeerJ. 2022 Sep 16;10:e13993. doi: 10.7717/peerj.13993 (PMC9484455; doi:10.7717/peerj.13993)

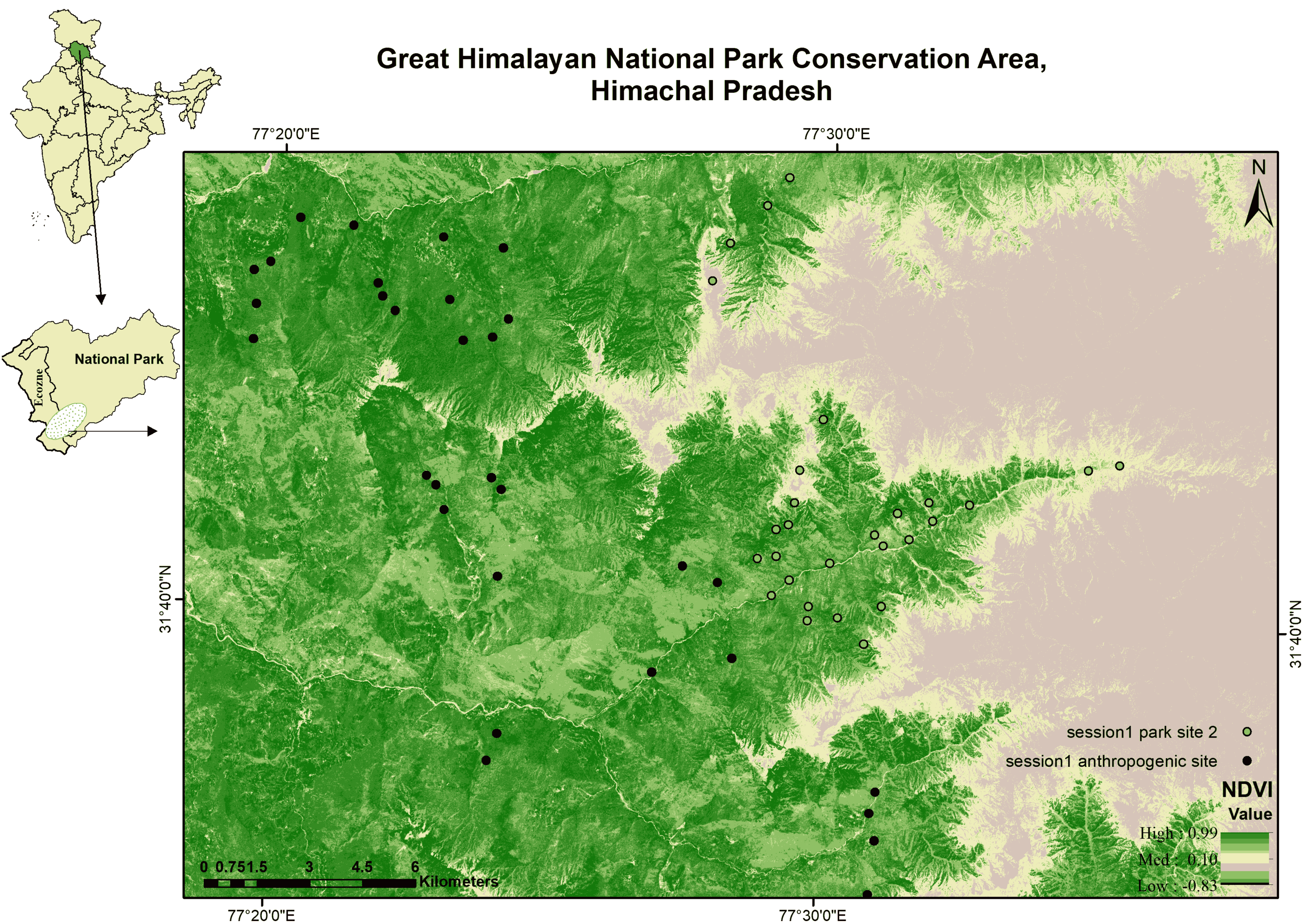

Supplement: Supplemental Information 1 [file peerj-10-13993-s001.png]

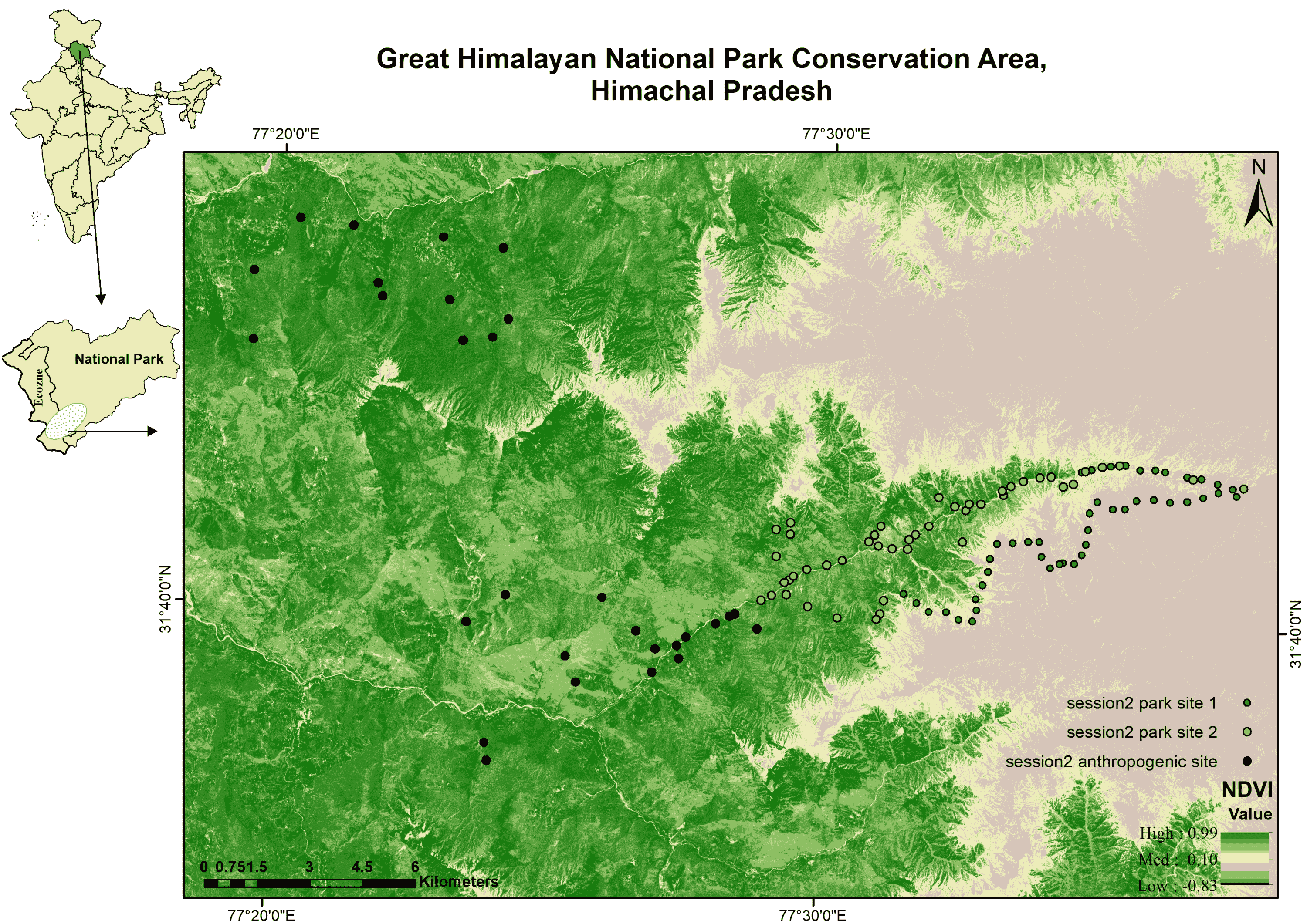

Supplement: Supplemental Information 2 [file peerj-10-13993-s002.png]

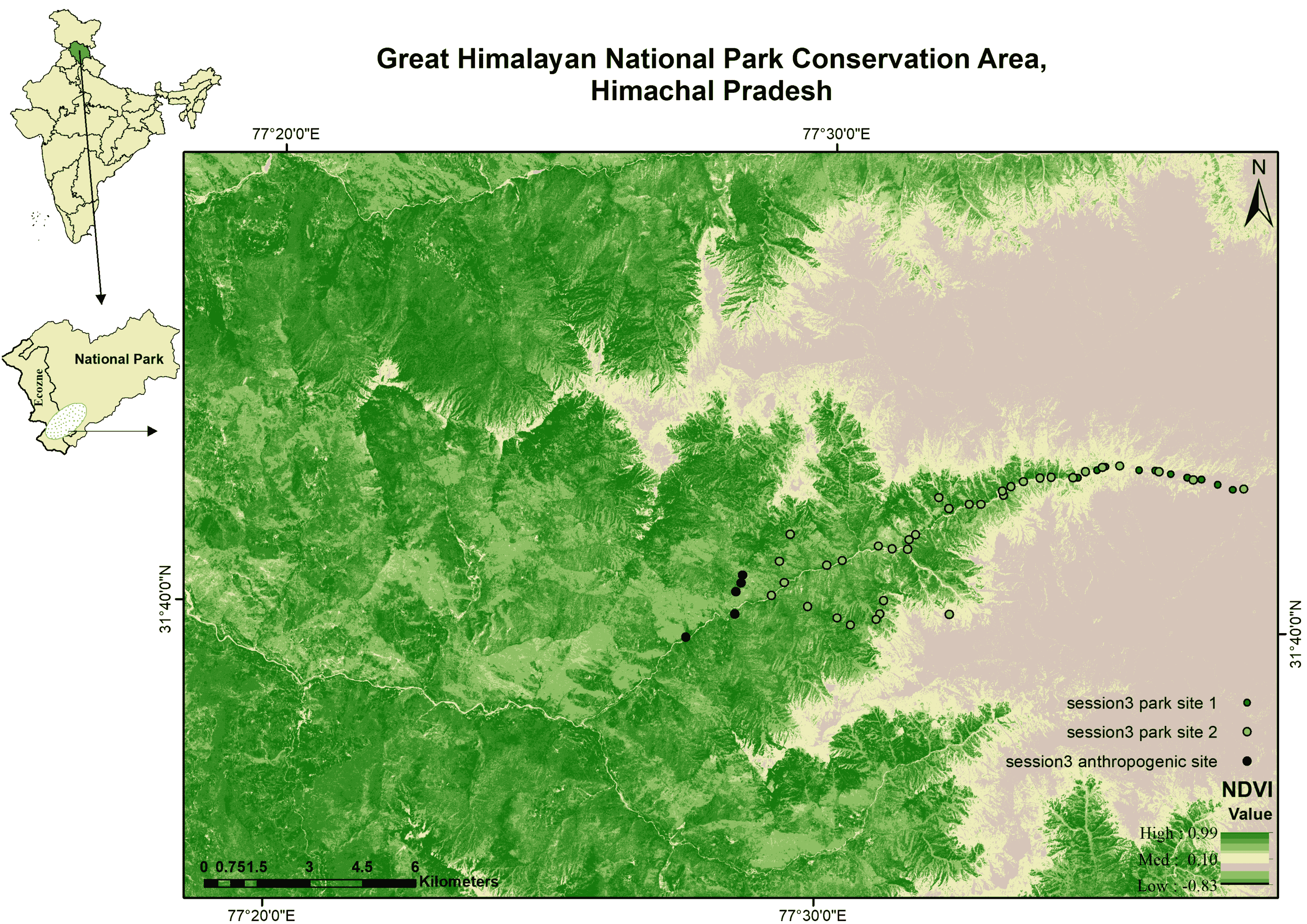

Supplement: Supplemental Information 3 [file peerj-10-13993-s003.png]

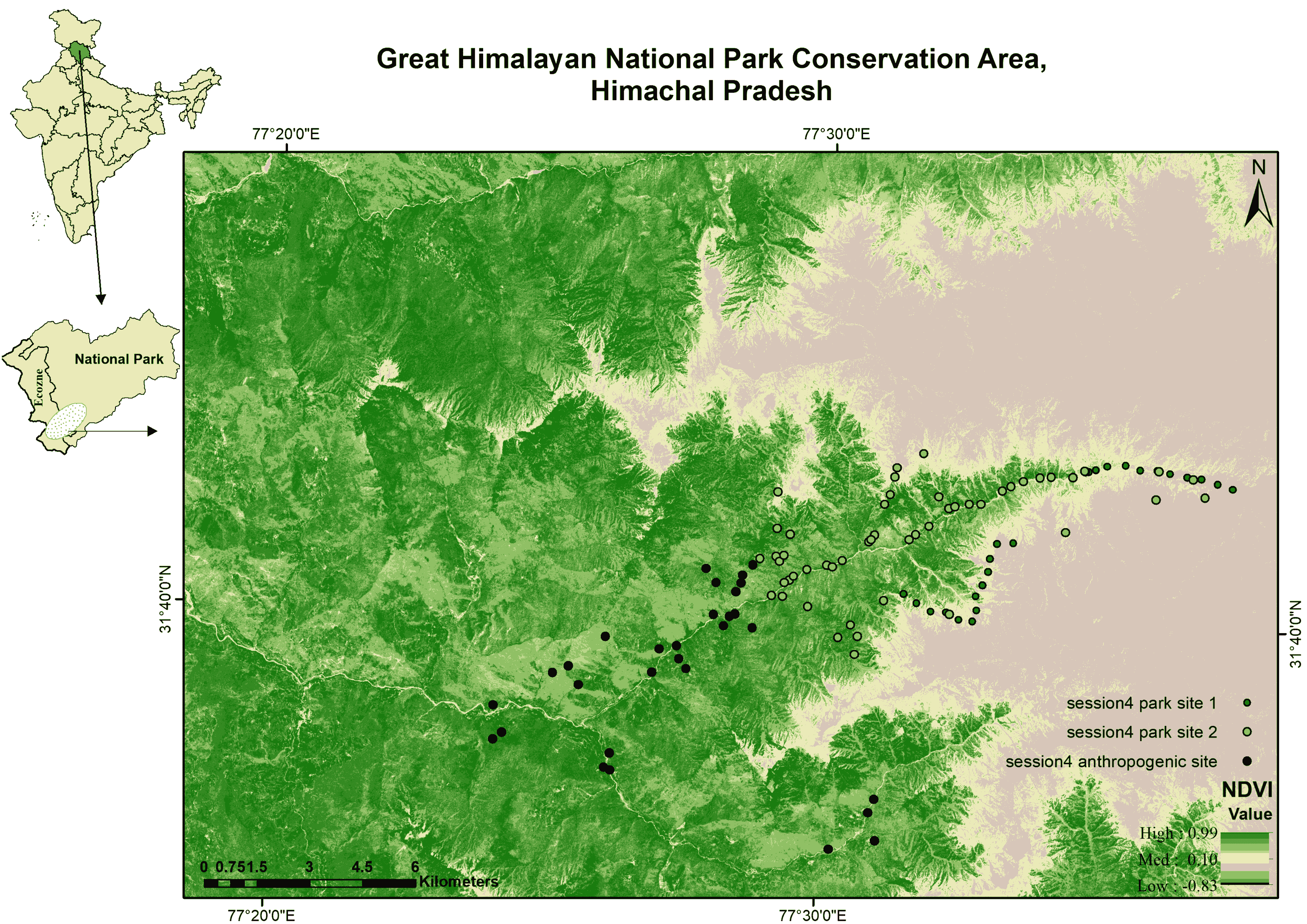

Supplement: Supplemental Information 4 [file peerj-10-13993-s004.png]

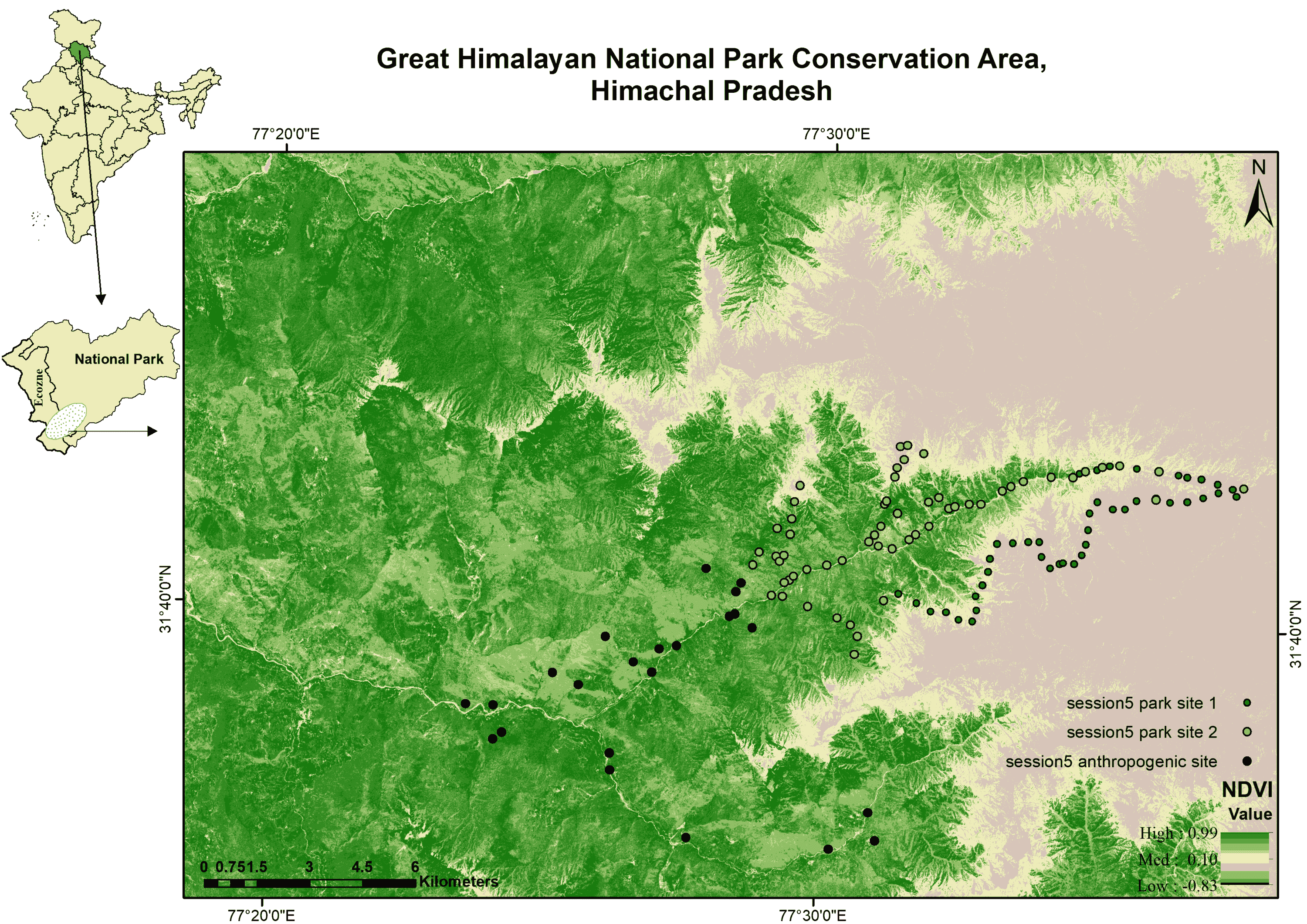

Supplement: Supplemental Information 5 [file peerj-10-13993-s005.png]

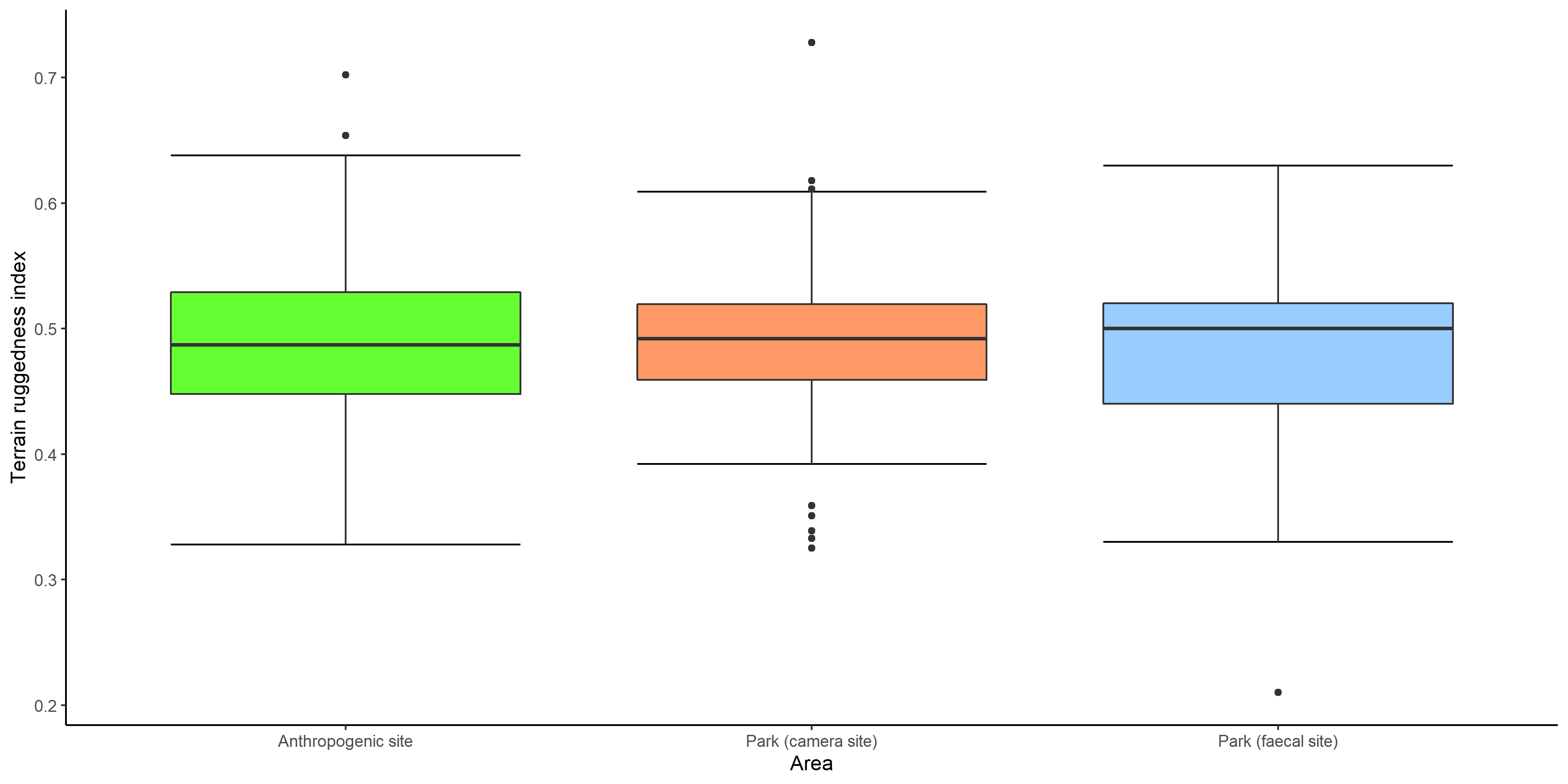

Supplement: Supplemental Information 6 [file peerj-10-13993-s006.png]

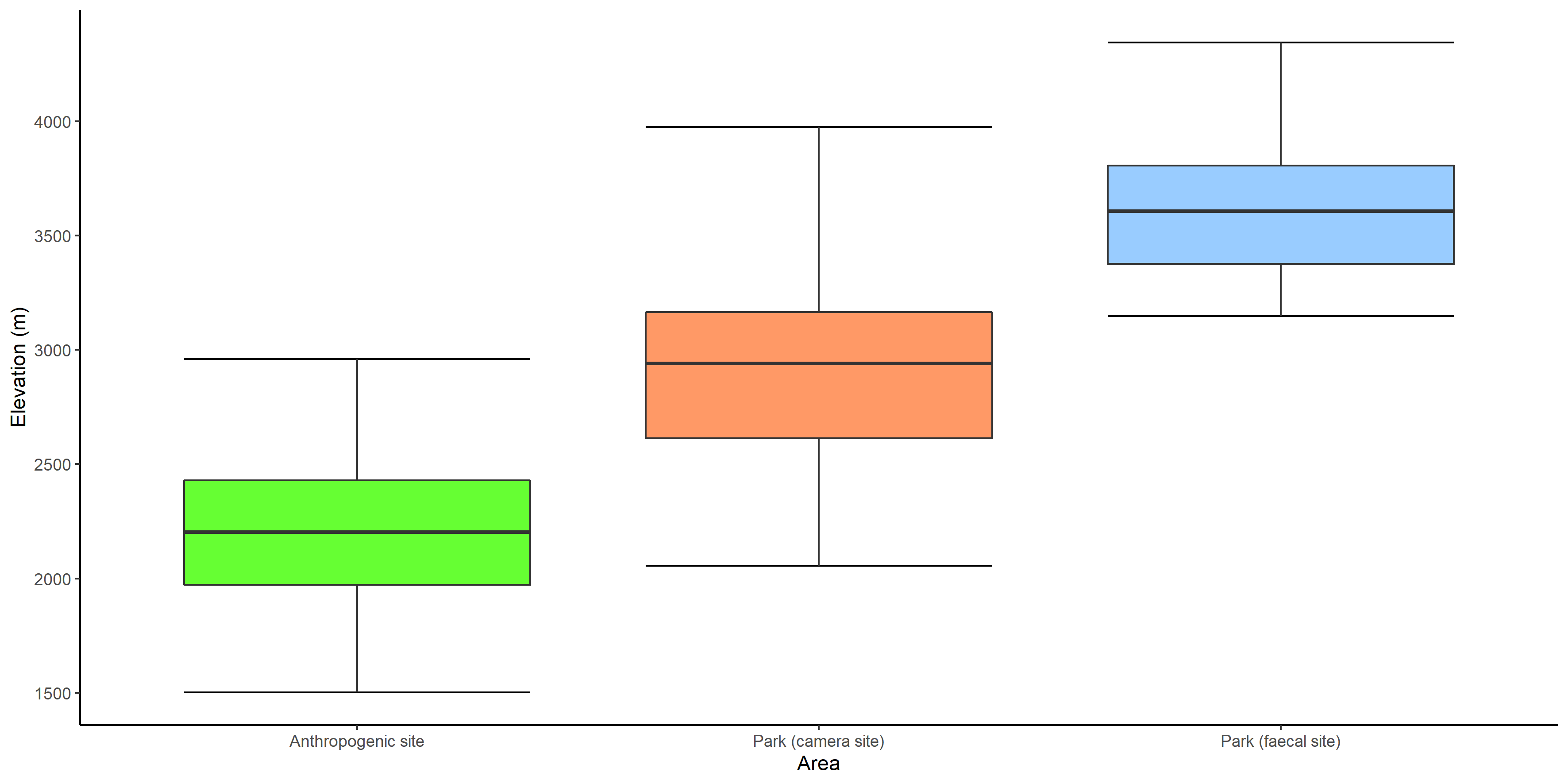

Supplement: Supplemental Information 7 [file peerj-10-13993-s007.png]

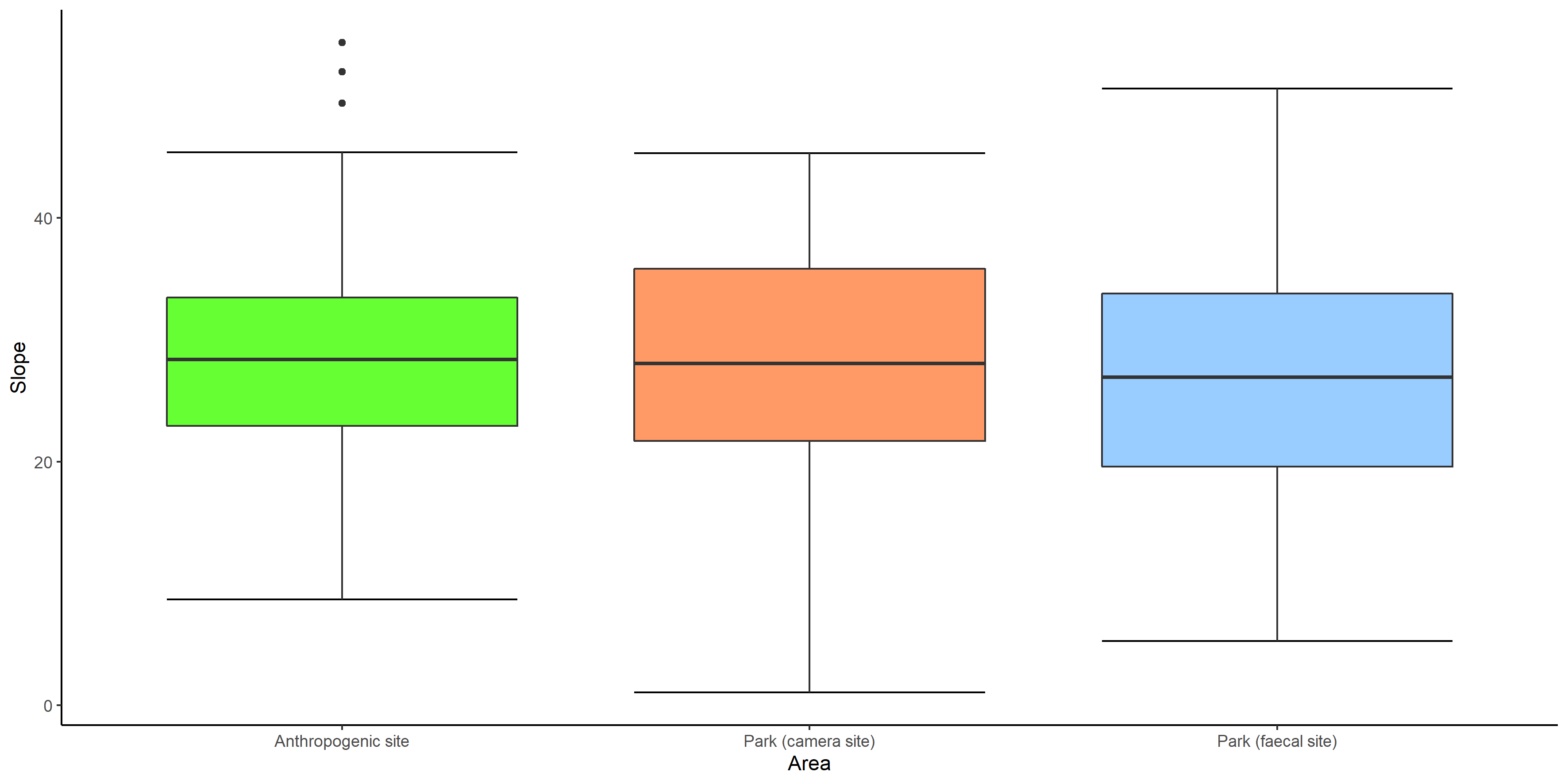

Supplement: Supplemental Information 8 [file peerj-10-13993-s008.png]

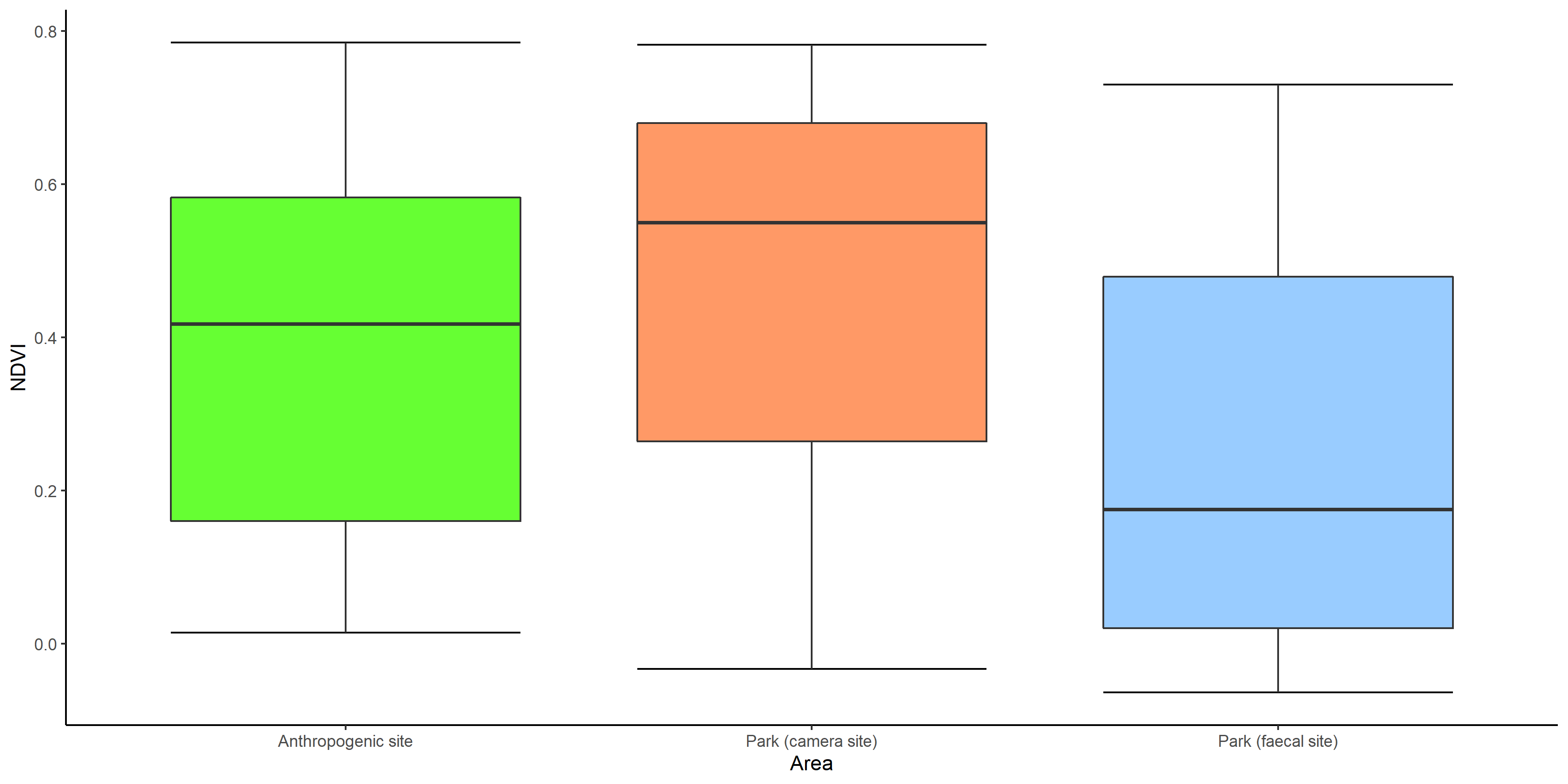

Supplement: Supplemental Information 9 [file peerj-10-13993-s009.png]

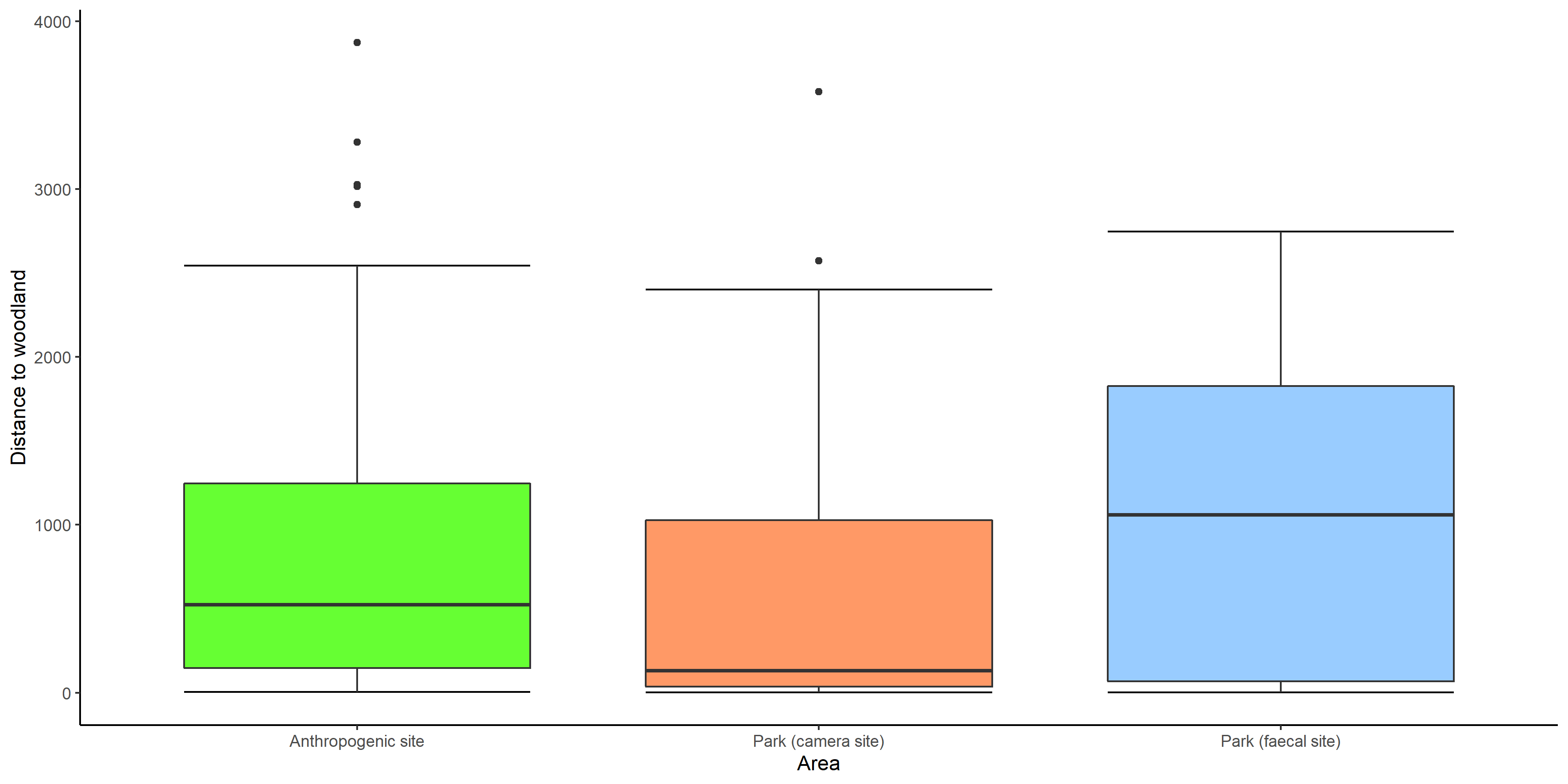

Supplement: Supplemental Information 10 [file peerj-10-13993-s010.png]

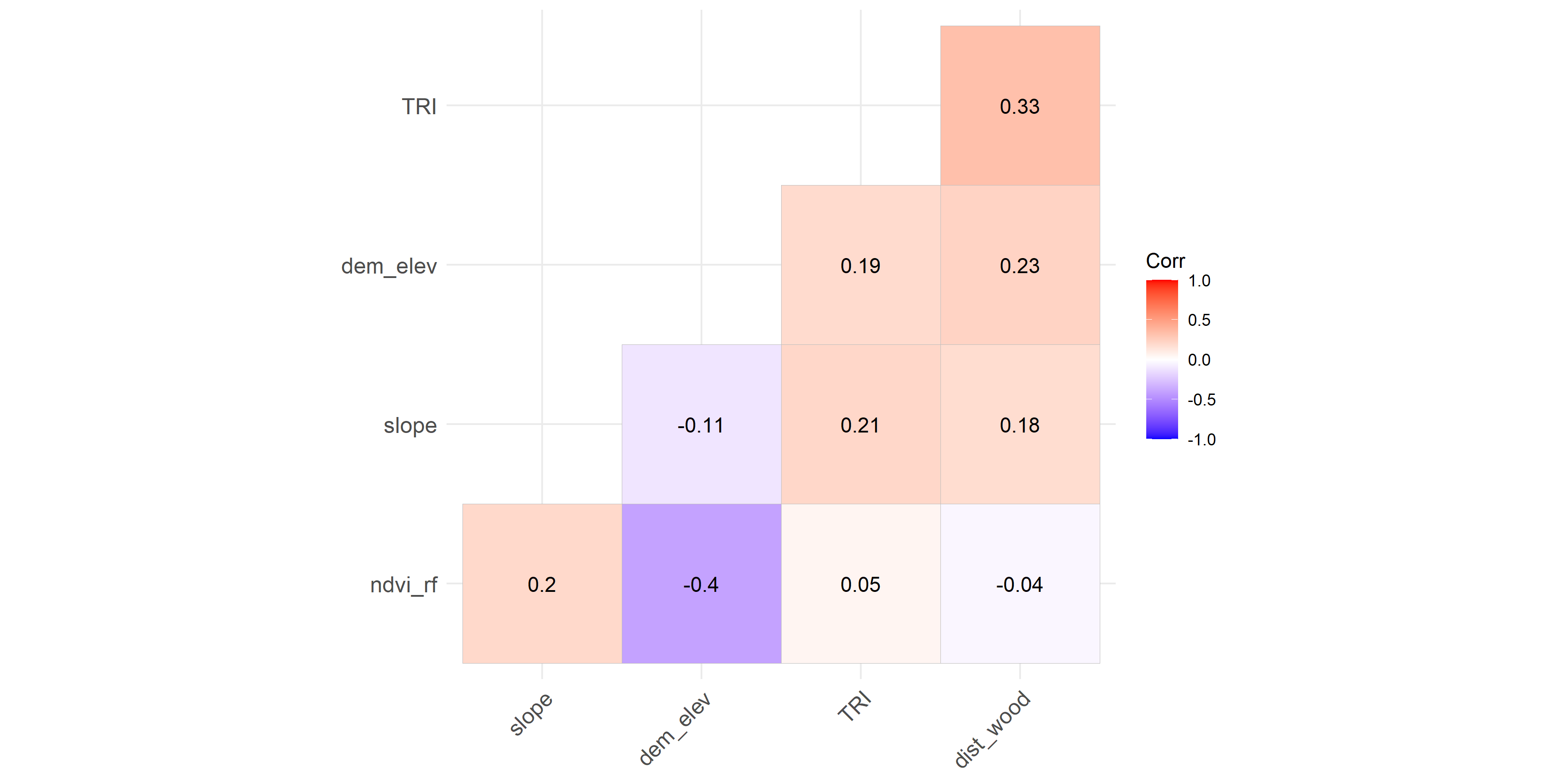

Supplement: Supplemental Information 11 [file peerj-10-13993-s011.png]

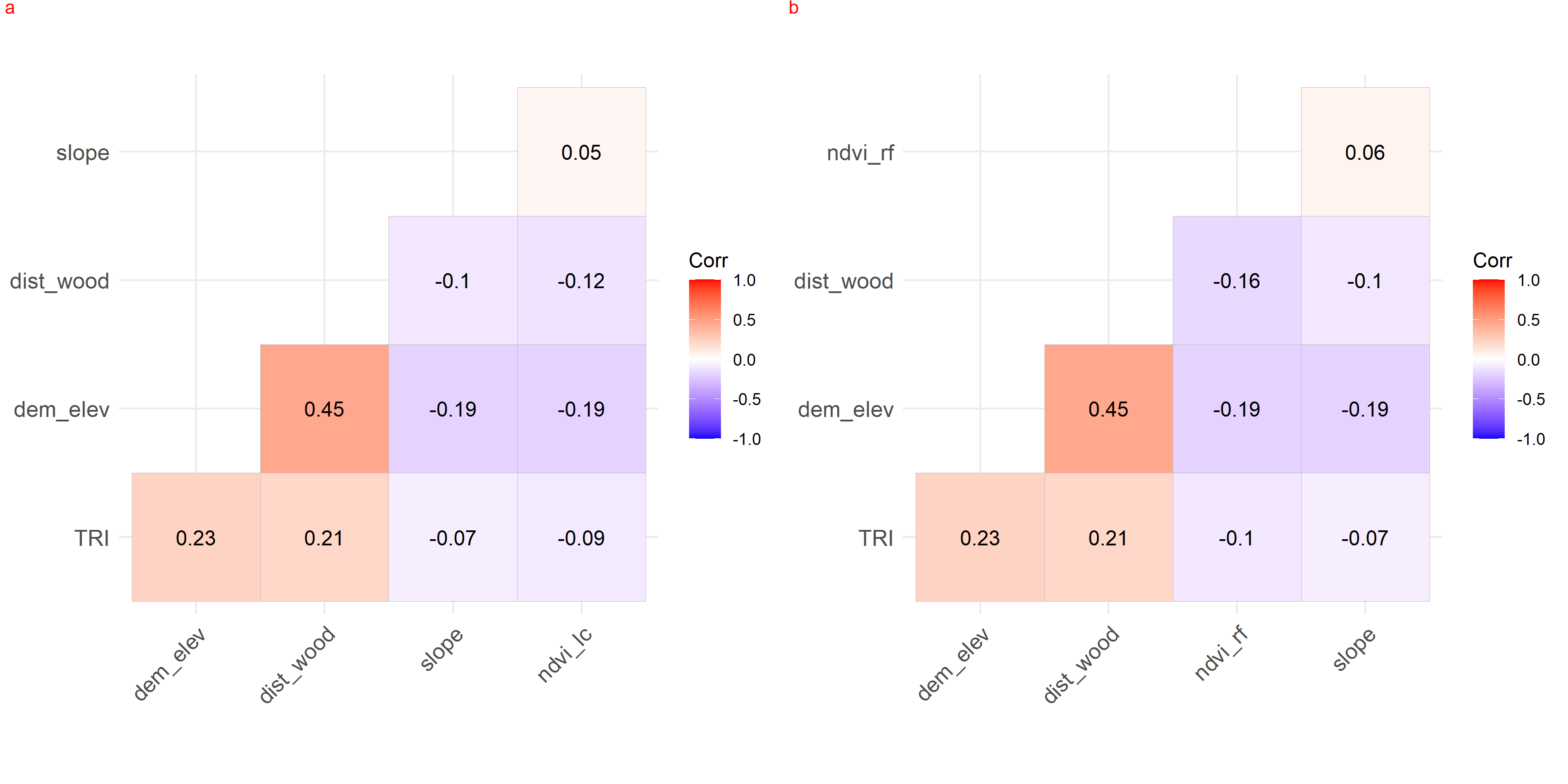

Supplement: Supplemental Information 12 [file peerj-10-13993-s012.png]

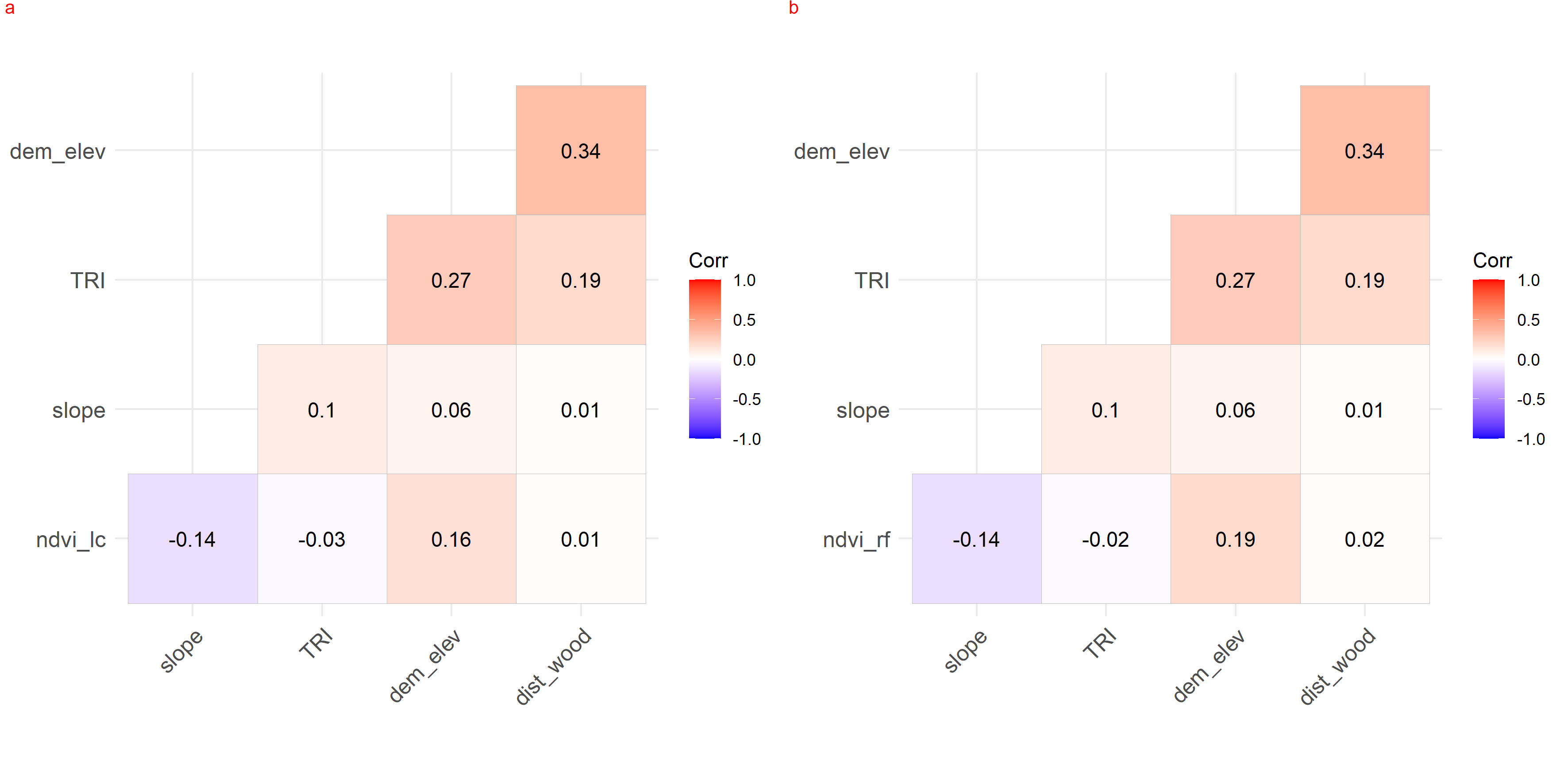

Supplement: Supplemental Information 13 [file peerj-10-13993-s013.png]

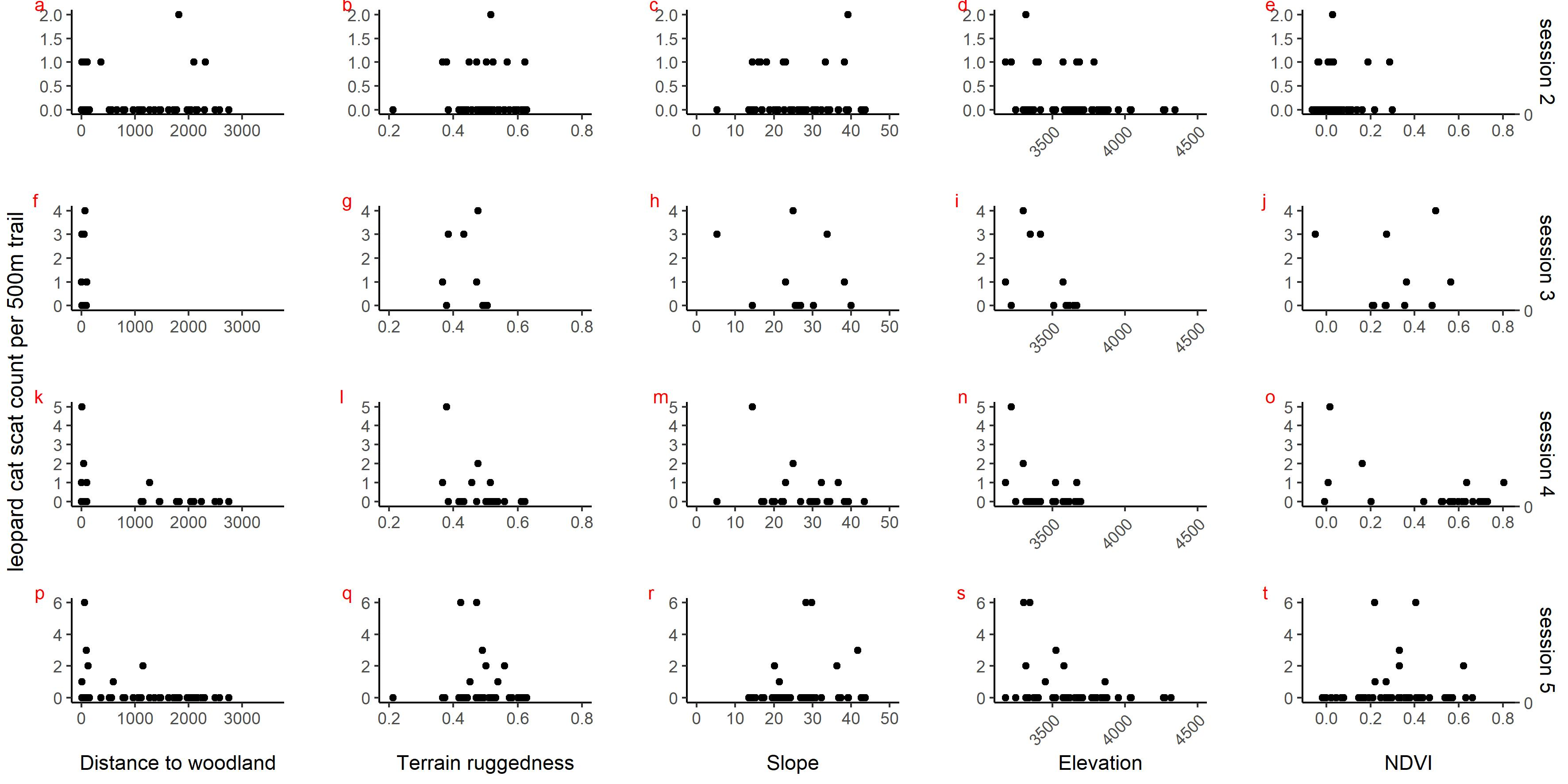

Supplement: Supplemental Information 14 [file peerj-10-13993-s014.png]

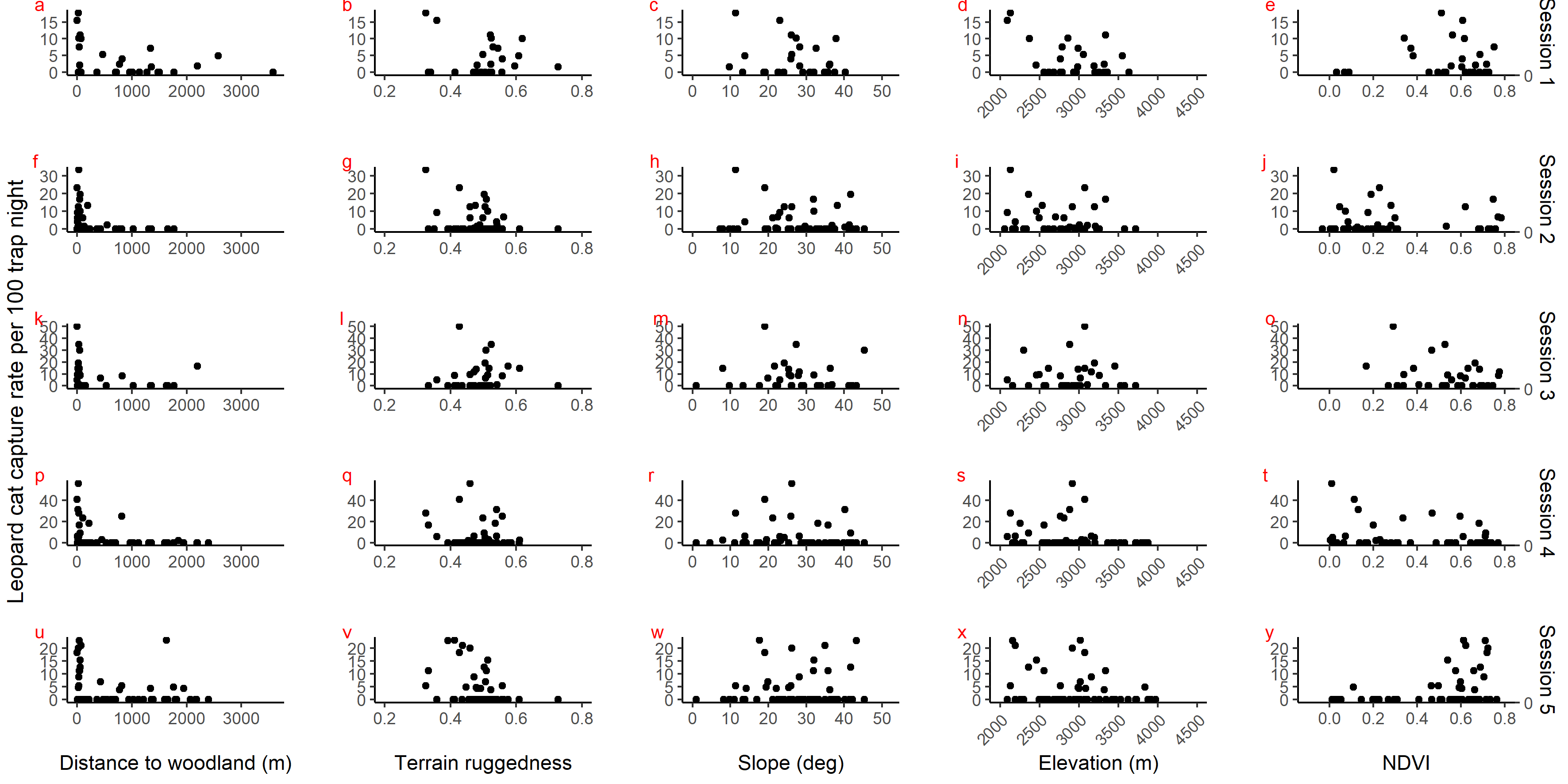

Supplement: Supplemental Information 15 [file peerj-10-13993-s015.png]

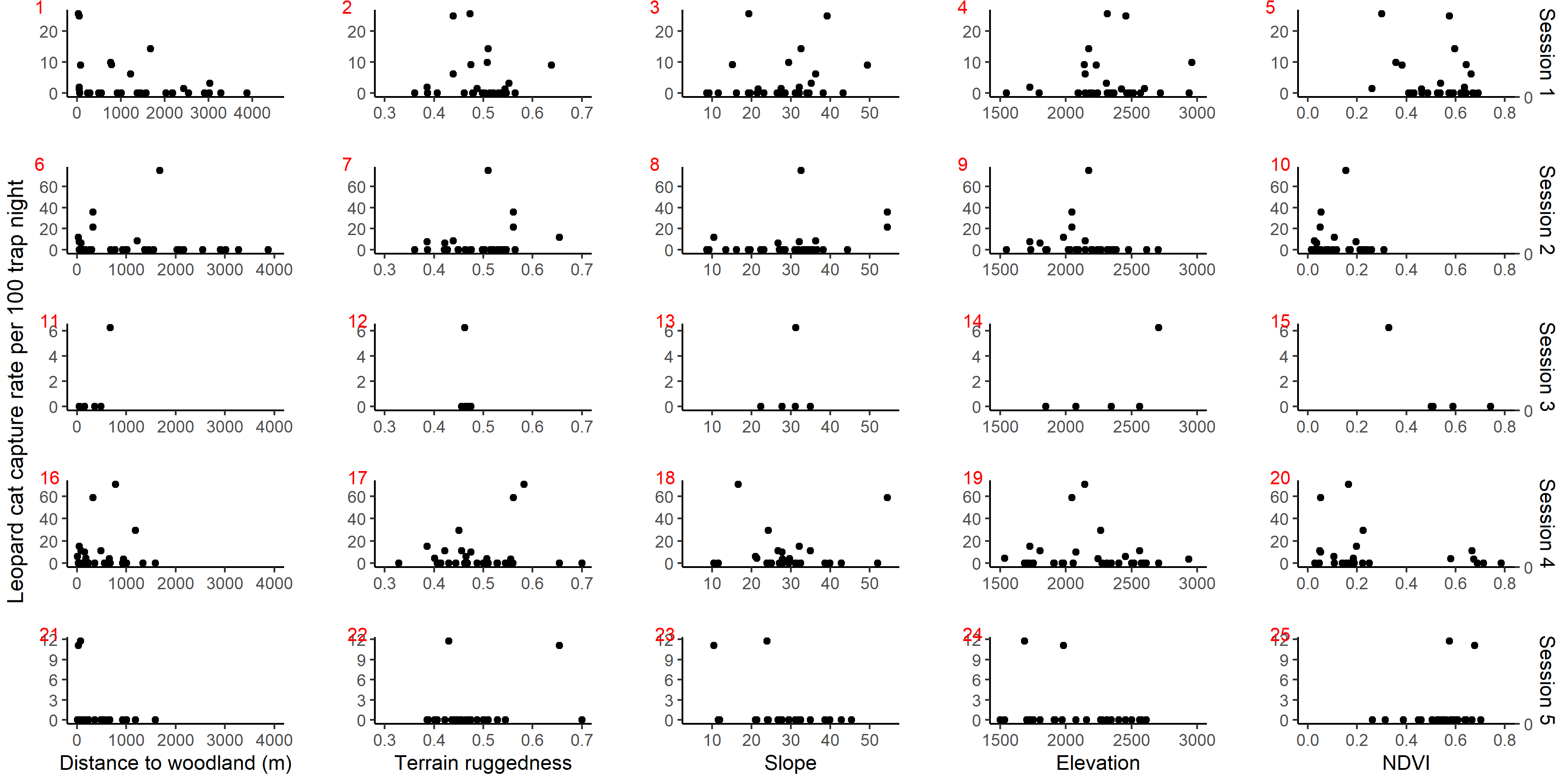

Supplement: Supplemental Information 16 [file peerj-10-13993-s016.png]

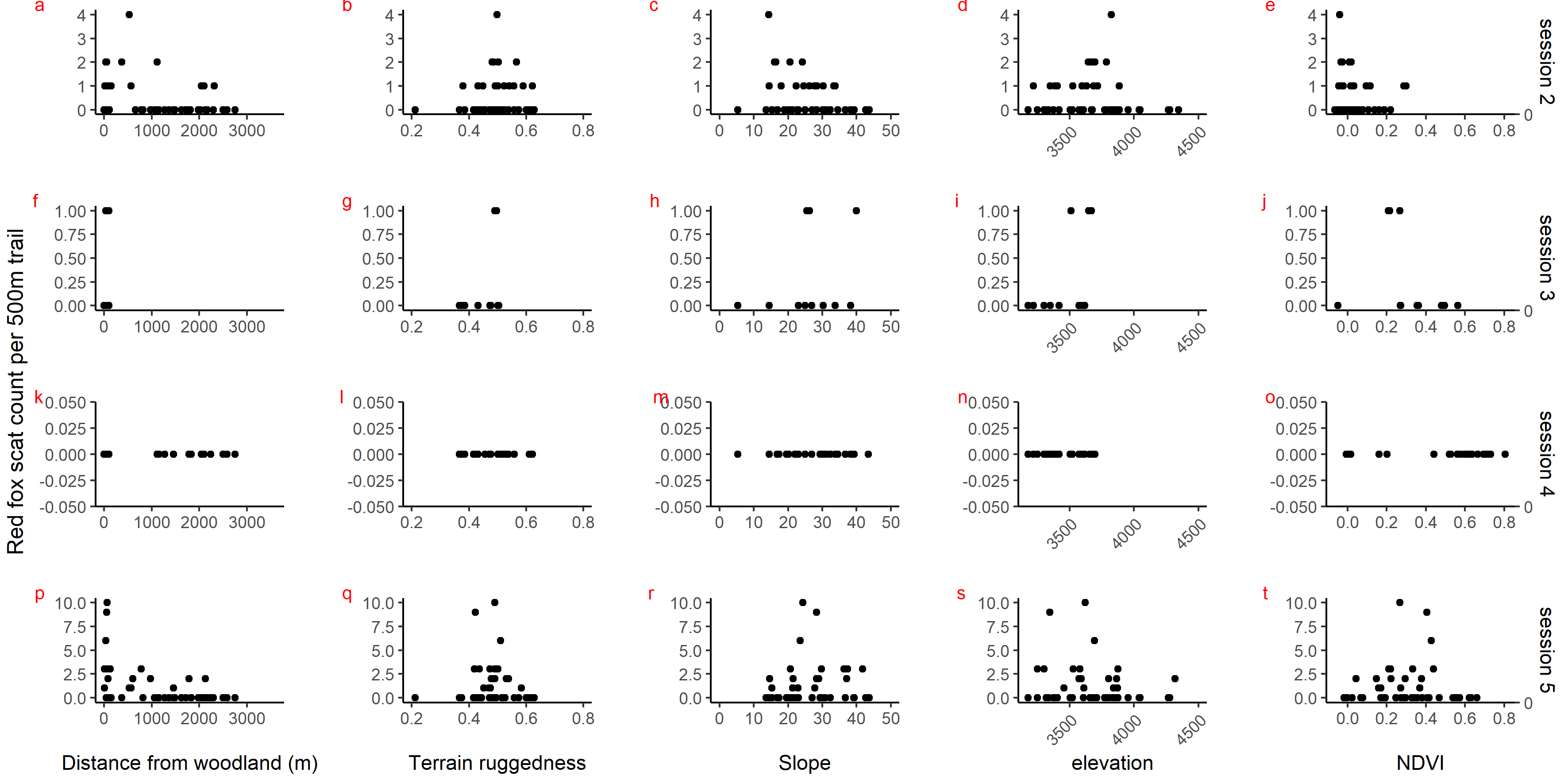

Supplement: Supplemental Information 17 [file peerj-10-13993-s017.png]

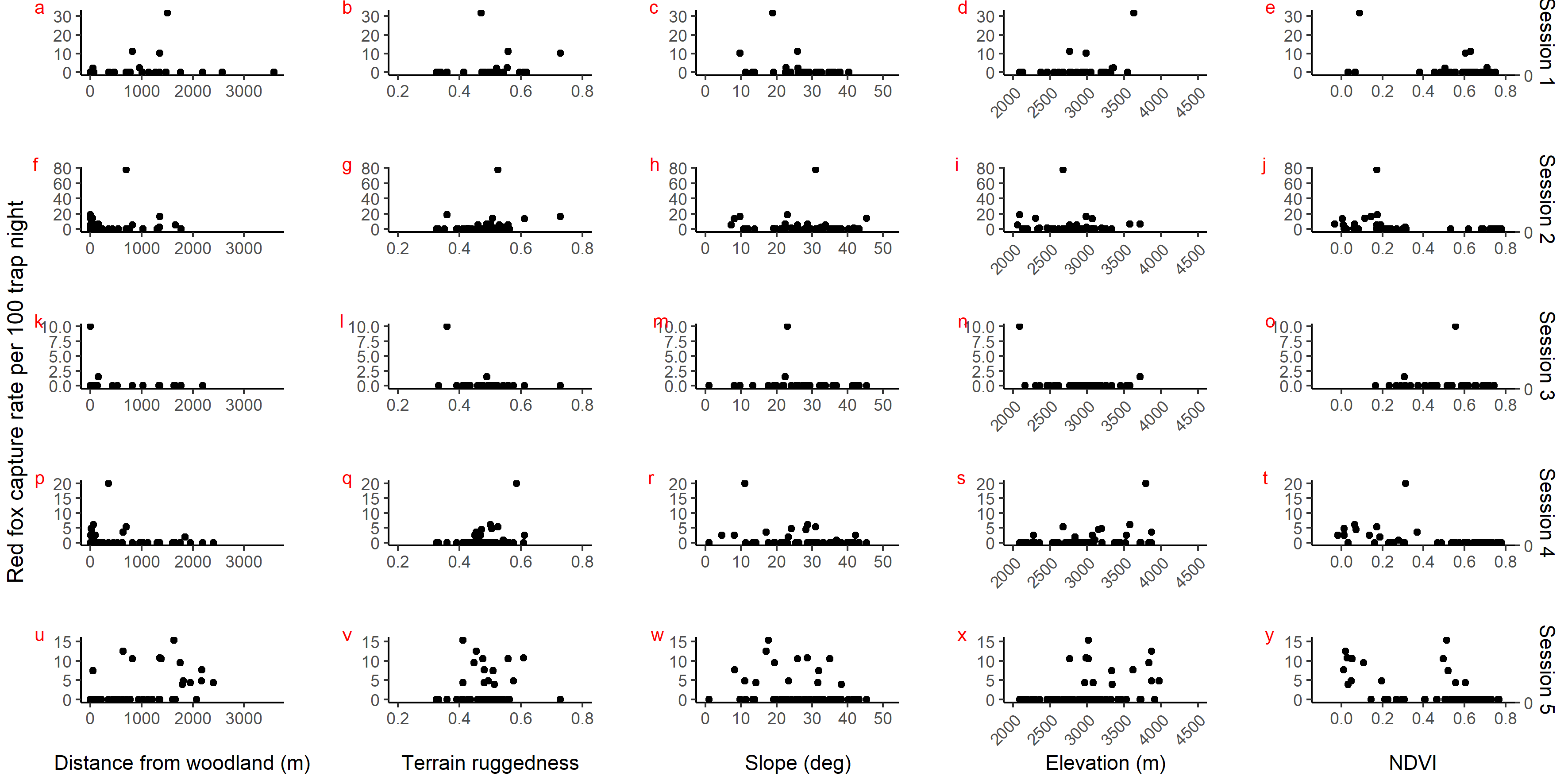

Supplement: Supplemental Information 18 [file peerj-10-13993-s018.png]

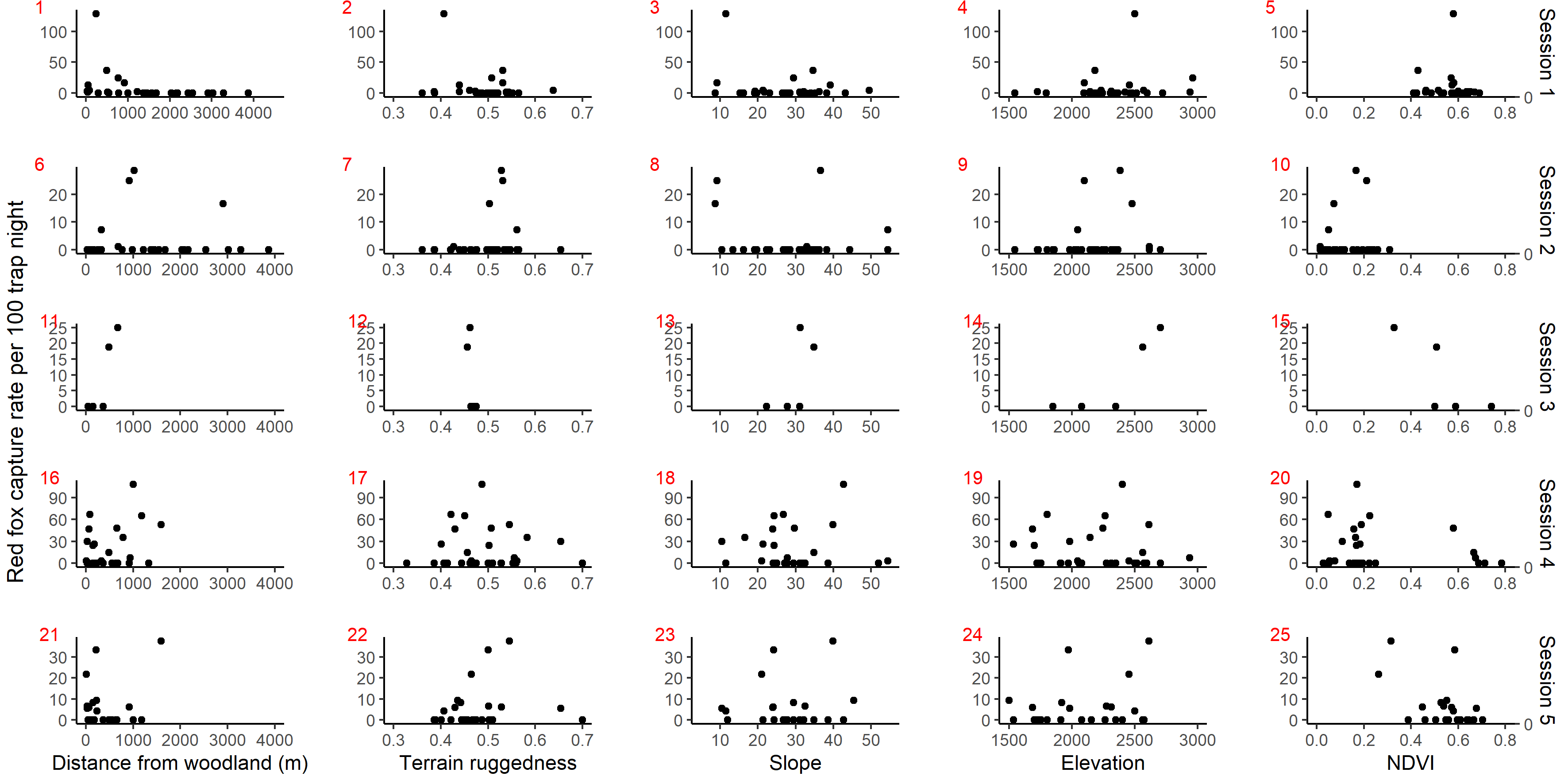

Supplement: Supplemental Information 19 [file peerj-10-13993-s019.png]

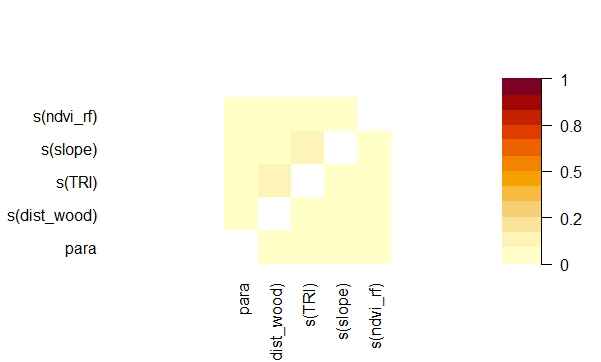

Supplement: Supplemental Information 20 [file peerj-10-13993-s020.png]

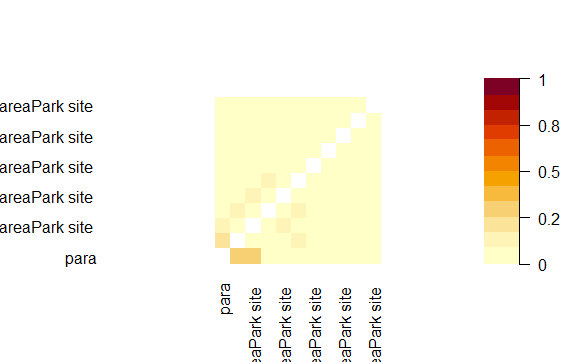

Supplement: Supplemental Information 21 [file peerj-10-13993-s021.png]

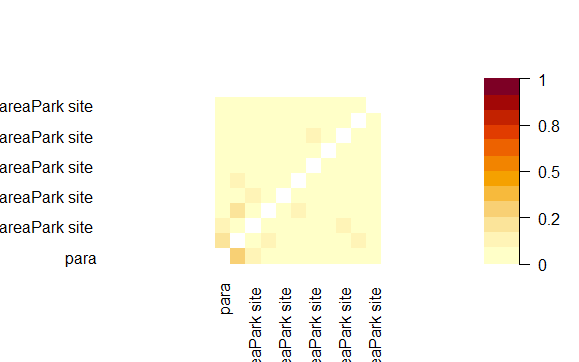

Supplement: Supplemental Information 22 [file peerj-10-13993-s022.png]

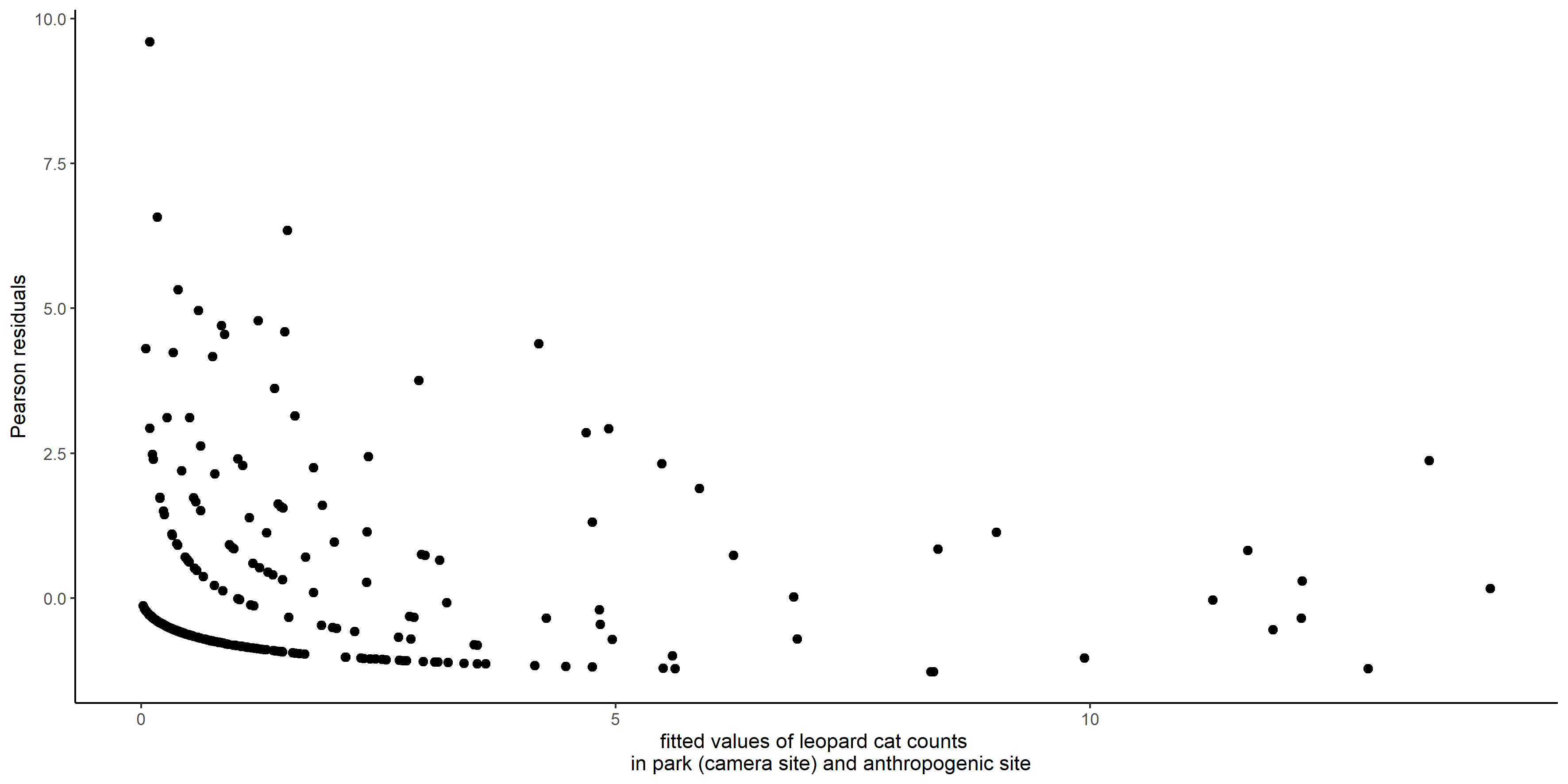

Supplement: Supplemental Information 23 [file peerj-10-13993-s023.png]

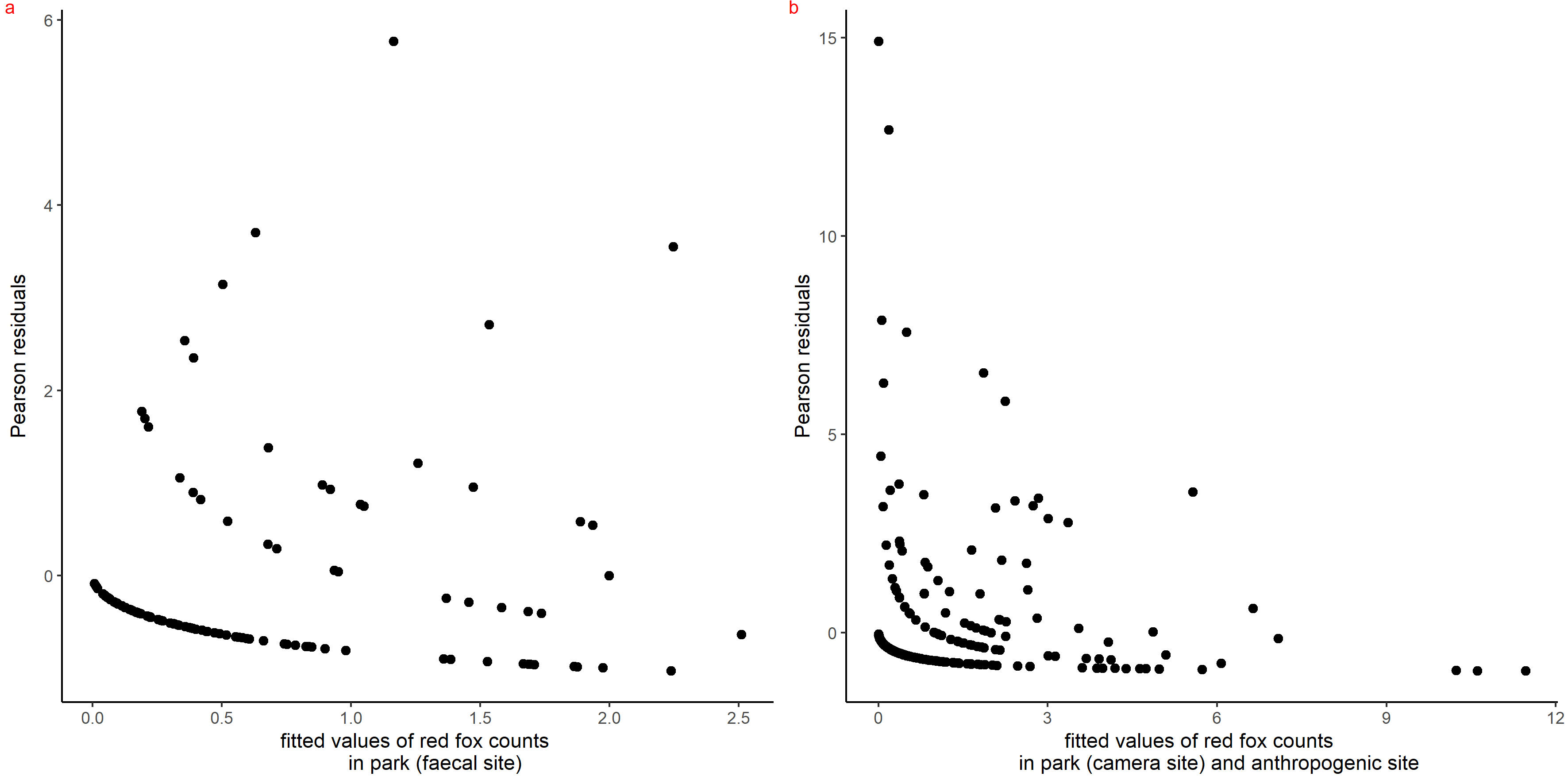

Supplement: Supplemental Information 24 [file peerj-10-13993-s024.png]

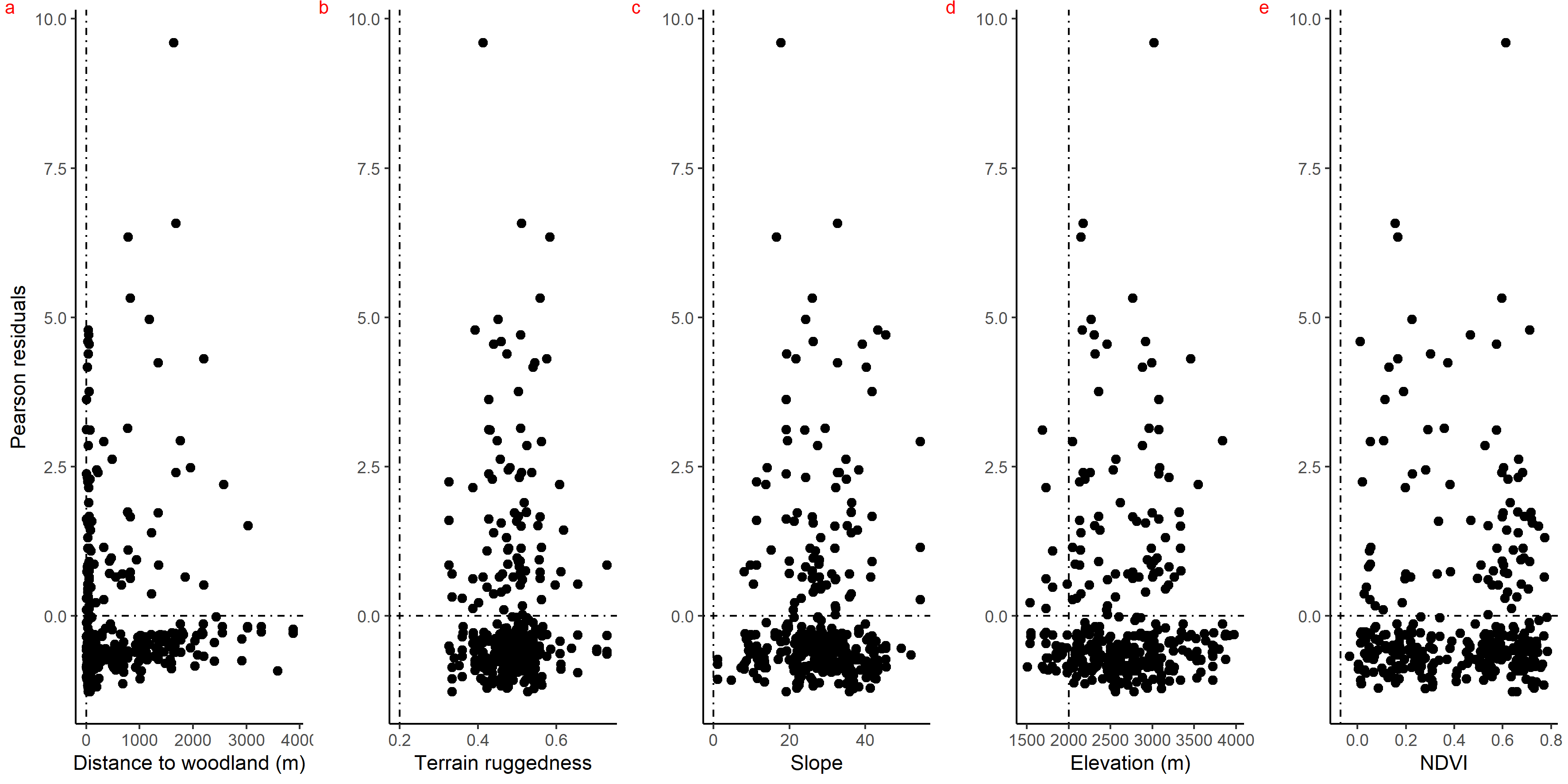

Supplement: Supplemental Information 25 [file peerj-10-13993-s025.png]

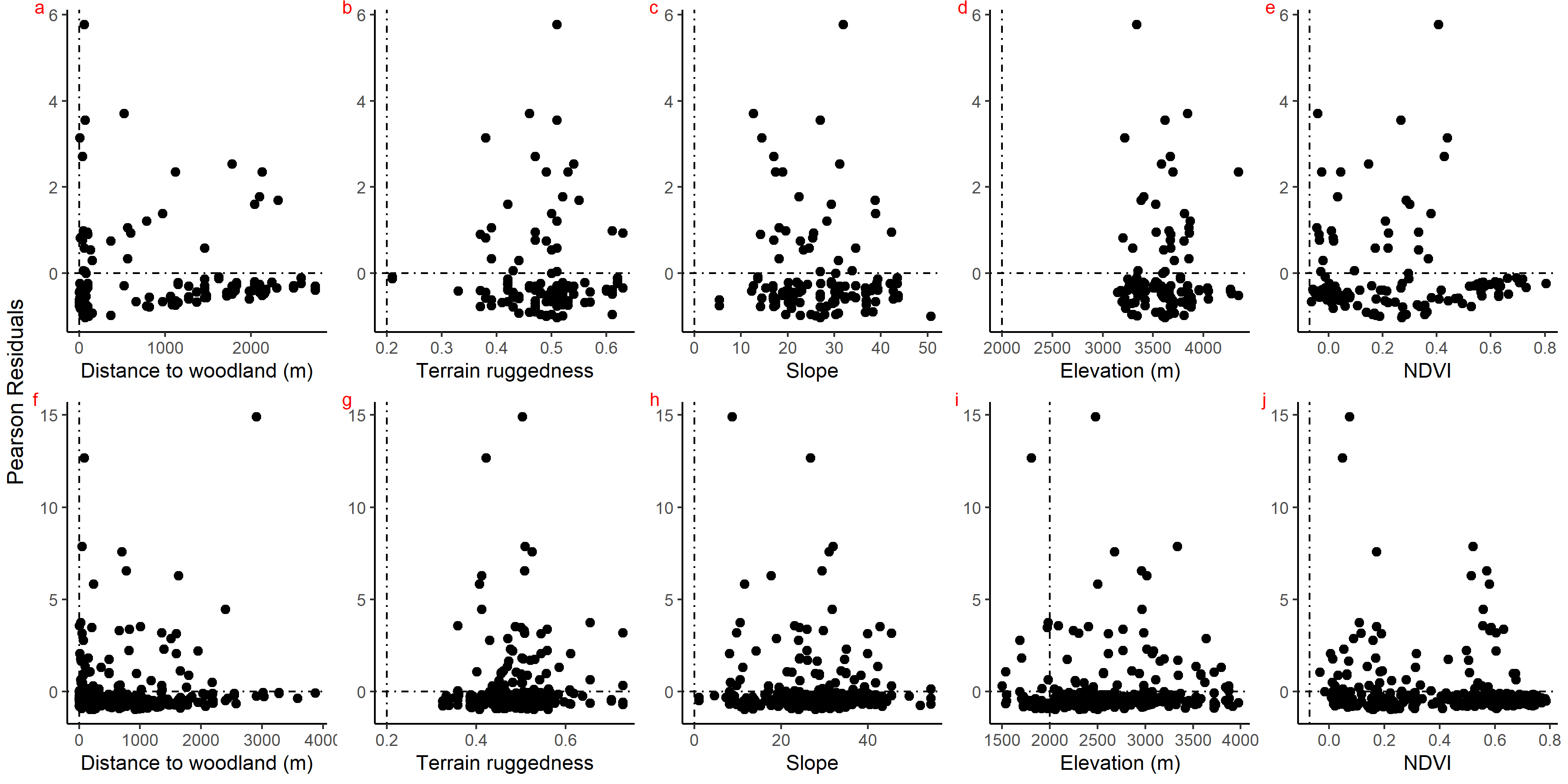

Supplement: Supplemental Information 26 [file peerj-10-13993-s026.png]

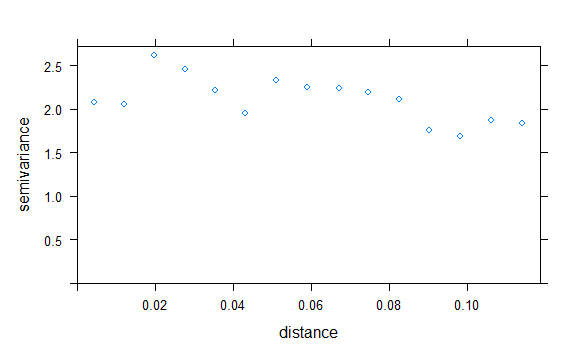

Supplement: Supplemental Information 27 [file peerj-10-13993-s027.png]

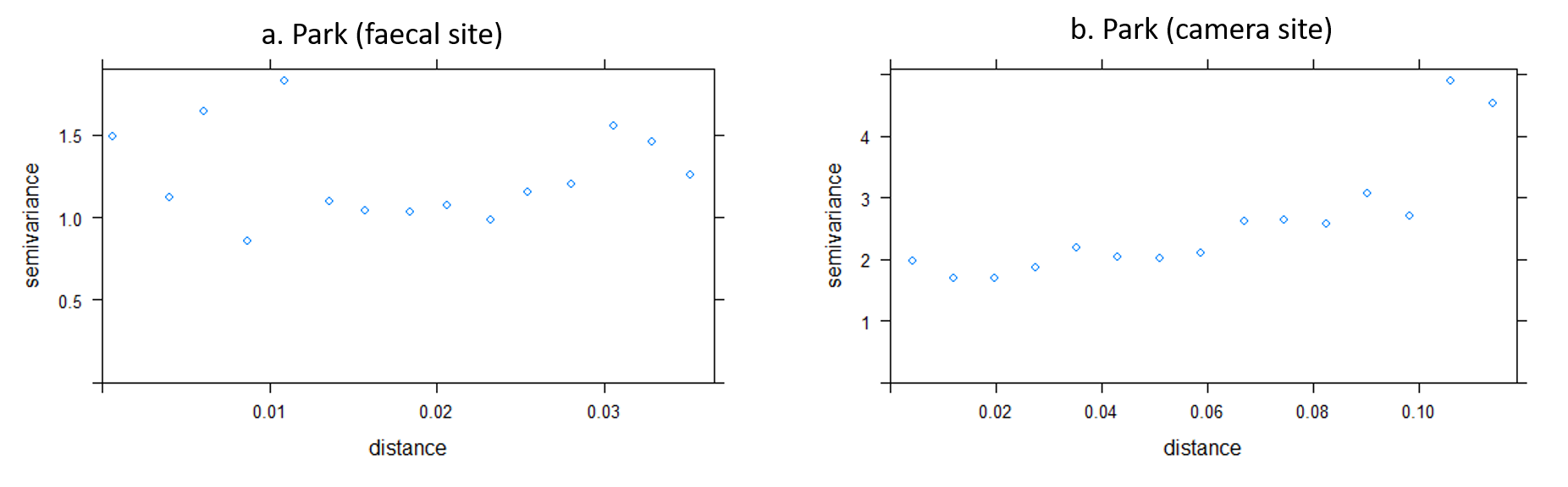

Supplement: Supplemental Information 28 [file peerj-10-13993-s028.png]

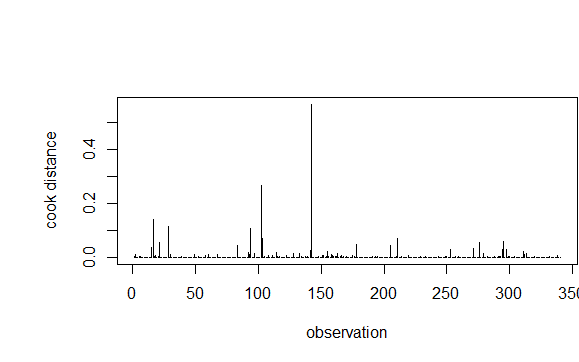

Supplement: Supplemental Information 29 [file peerj-10-13993-s029.png]

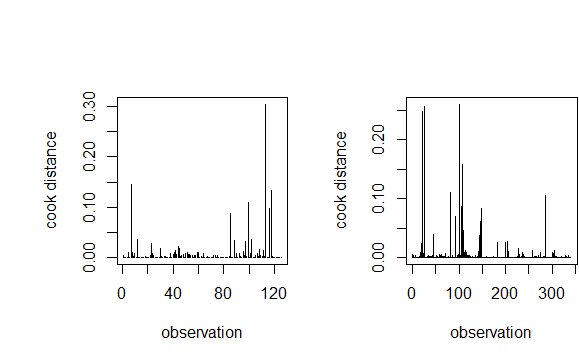

Supplement: Supplemental Information 30 [file peerj-10-13993-s030.png]

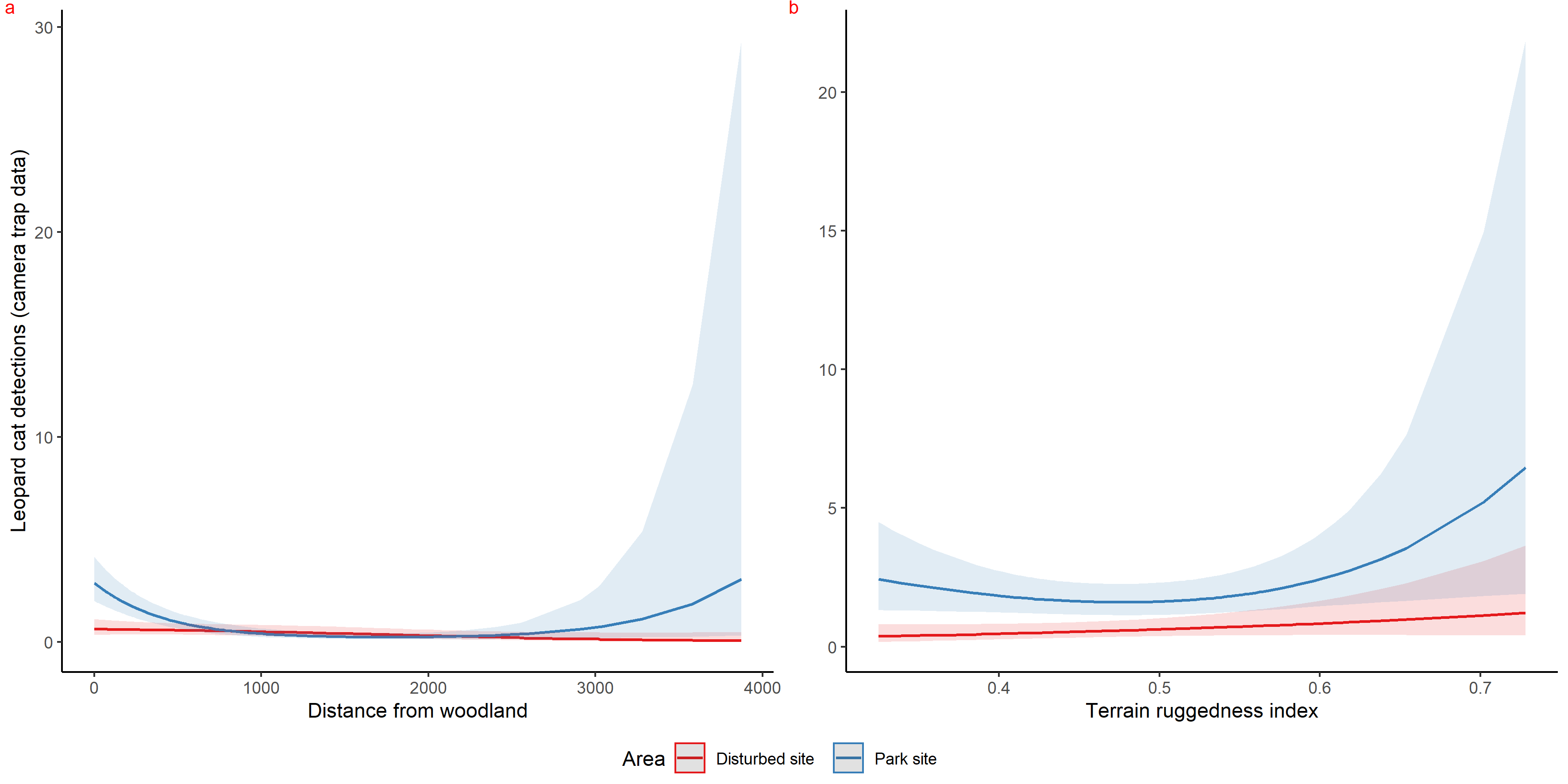

Supplement: Supplemental Information 31 [file peerj-10-13993-s031.png]

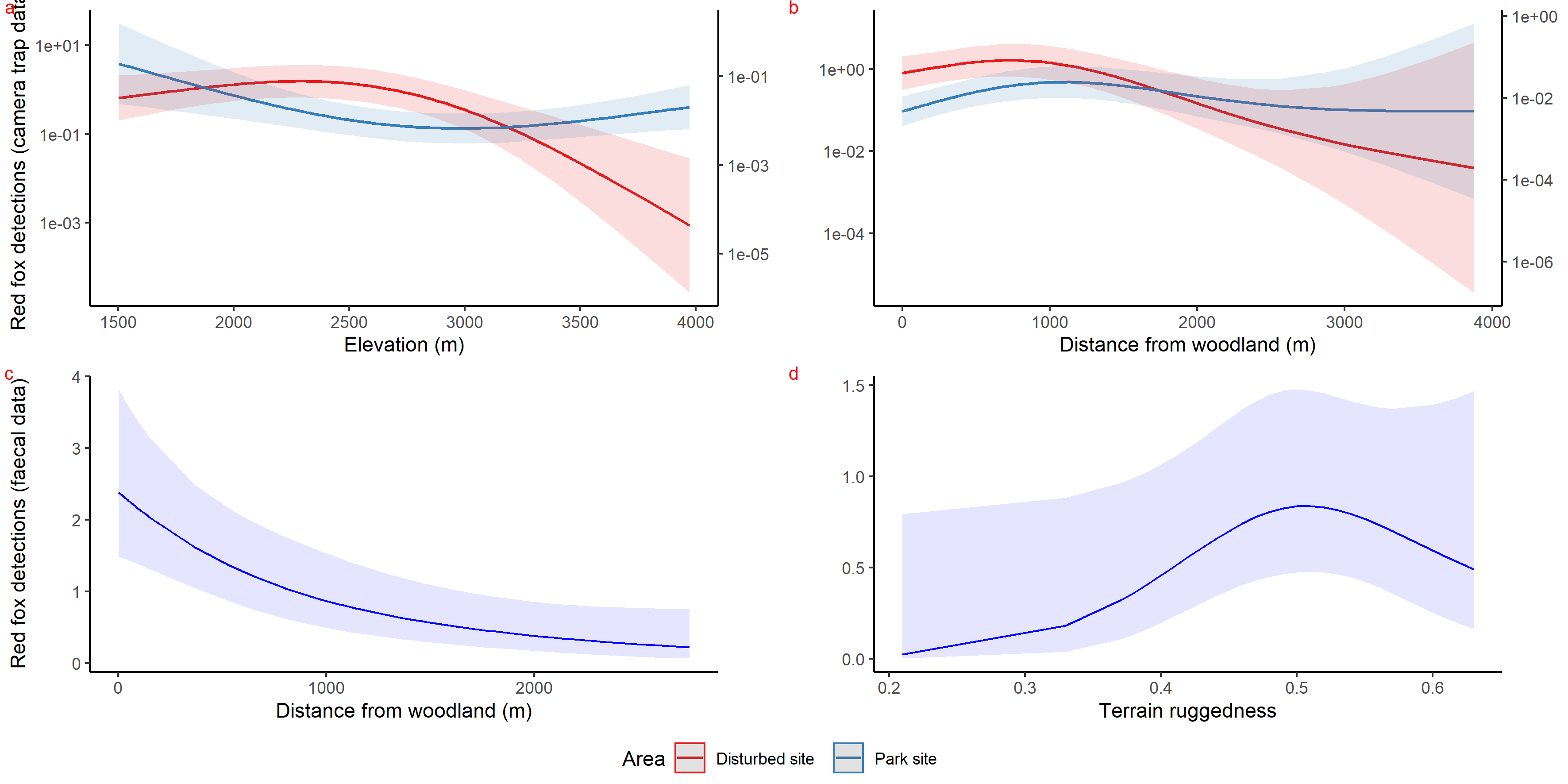

Supplement: Supplemental Information 32 [file peerj-10-13993-s032.png]

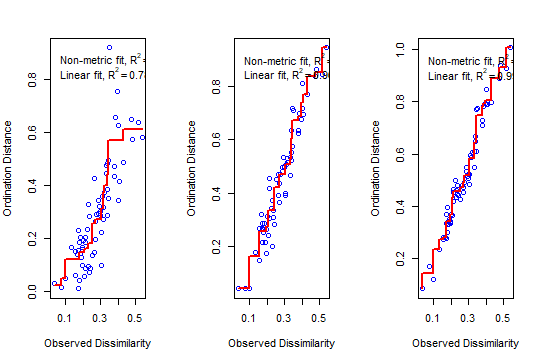

Supplement: Supplemental Information 33 [file peerj-10-13993-s033.png]

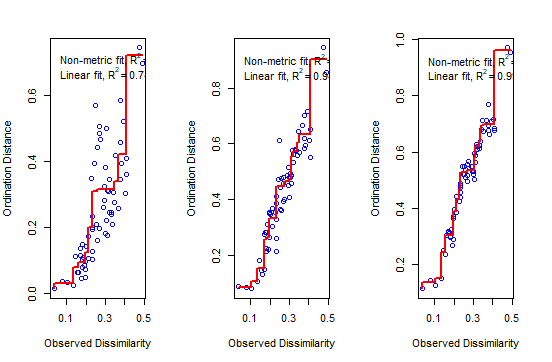

Supplement: Supplemental Information 34 [file peerj-10-13993-s034.png]
