# Supplementary material for: Understanding the distribution and fine-scale habitat selection of mesocarnivores along a habitat quality gradient in western Himalaya
Source: PeerJ. 2022 Sep 16;10:e13993. doi: 10.7717/peerj.13993 (PMC9484455; doi:10.7717/peerj.13993)
Supplement: Supplemental Information 35 — Park site 1 shows number of surveyed trails for carnivore faecal sample collection, Park site 2 and anthropogenic site indicates number of camera trap location [file peerj-10-13993-s035.docx]

Table S1:

Session wise sampling details (camera trapping and faces collection) in GHNPCA during 2017 to 2019. Park site 1 shows number of surveyed trails for carnivore faecal sample collection, park site 2 and anthropogenic site indicates number of camera trap locations.

| Session | Month | Year | Number of trails surveyed | Number of camera trap locations | | Range of no. of camera trap days for each camera trap | | Effort | | |
| --- | --- | --- | --- | --- | --- | --- | --- | --- | --- | --- |
|  |  |  | Park site 1  (national park) | Park site 2  (national park) | Anthropogenic site  (ecozone) | Park site 2  (national park) | Anthropogenic site  (ecozone) | Park site 1:  Distance walked (km)  (national park) | Park site 2:  Sum of no. of days of all camera traps  (national park) | Anthropogenic site:  Sum of no. of days of all camera traps  (ecozone) |
| 1 | April-July | 2017 | 0 | 28 | 31 | 8 to 127 | 5 to 78 | 0 | 1907 | 1079 |
| 2 | October-December | 2017 | 45 | 47 | 31 | 4 to 211 | 2 to 92 | 22.5 | 2189 | 400 |
| 3 | April-July | 2018 | 11 | 35 | 5 | 6 to 122 | 14 to 35 | 5.5 | 1694 | 97 |
| 4 | October-December | 2018 | 24 | 52 | 30 | 4 to 134 | 2 to 54 | 12.0 | 2125 | 612 |
| 5 | April-June | 2019 | 45 | 58 | 23 | 2 to 180 | 3 to 33 | 22.5 | 1369 | 394 |
|  |  | Total | 125 | 220 | 120 |  |  |  | 9284 | 2582 |
