# Supplementary material for: Understanding the distribution and fine-scale habitat selection of mesocarnivores along a habitat quality gradient in western Himalaya
Source: PeerJ. 2022 Sep 16;10:e13993. doi: 10.7717/peerj.13993 (PMC9484455; doi:10.7717/peerj.13993)
Supplement: Supplemental Information 36 [file peerj-10-13993-s036.docx]

Table S2:

Details of explanatory variables used in generalised additive modelling for red fox and leopard cat.

| Species | Mesocarnivore detections best explained by | Predictor variables | Data acquisition layer | Values range |
| --- | --- | --- | --- | --- |
| Mesocarnivore in anthropogenic site | Habitat variables | Elevation | Digital elevation model (DEM) | 1512m to 2946m |
|  |  | Terrain ruggedness index (TRI) | Digital elevation model (DEM) | 0.33 to 0.65 |
|  |  | slope | Digital elevation model (DEM) | 8.68° to 54.42° |
|  |  | Normalized difference vegetation index (NDVI) | Sentinel 2: April to July and October to December | 0.01 to 0.78 |
|  |  | Distance from woodland | River shapefile: Distance from main river bed to each camera trap location | 4m to 3874m |
| Mesocarnivore in park site 2 | Habitat variables | Elevation | Digital elevation model (DEM) | 2055m to 3974m |
|  |  | Terrain ruggedness index (TRI) | Digital elevation model (DEM) | 0.32 to 0.73 |
|  |  | slope | Digital elevation model (DEM) | 1.04° to 45.34° |
|  |  | Normalized difference vegetation index (NDVI) | Sentinel 2: April to July and October to December | -0.03 to 0.78 |
|  |  | Distance from woodland | River shapefile: Distance from main river bed to each camera trap location | 1m to 3581m |
| Mesocarnivore in park site 1 | Habitat variables | Elevation | Digital elevation model (DEM) | 3148m to 4345m |
|  |  | Terrain ruggedness index (TRI) | Digital elevation model (DEM) | 0.21 to 0.63 |
|  |  | slope | Digital elevation model (DEM) | 5.28° to 50.64° |
|  |  | Normalized difference vegetation index (NDVI) | Sentinel 2: April to July and October to December | -0.06 to 0.73 |
|  |  | Distance from woodland | River shapefile: Distance from main river bed to each camera trap location | 1m to 2746m |
