# Supplementary material for: Understanding the distribution and fine-scale habitat selection of mesocarnivores along a habitat quality gradient in western Himalaya
Source: PeerJ. 2022 Sep 16;10:e13993. doi: 10.7717/peerj.13993 (PMC9484455; doi:10.7717/peerj.13993)
Supplement: Supplemental Information 38 — Village proximity refers to distance of camera trap locations from village centroids (within; 0–300 m, near; 300–600 m, away; >600 m). [file peerj-10-13993-s038.docx]

Table S4:

RAI (capture rate per 100 trap night) of leopard cat and human disturbance variables (human, dog, livestock) in anthropogenic site (ecozone) in GHNPCA used in NMDS. Village proximity refers to distance of camera trap locations from village centroids (within; 0m-300m, near; 300m-600m, away; >600m).

| Species | Village proximity | Agricultural plot | House | Village trail | Hill base | Hill slope | Hill top |
| --- | --- | --- | --- | --- | --- | --- | --- |
| leopard cat | within | 14.62 | 6.47 | 2.73 | 8.83 | 9.80 | 0.00 |
| human | within | 171.02 | 619.87 | 331.69 | 414.54 | 92.16 | 0.00 |
| Dog | within | 10.97 | 33.26 | 13.11 | 22.01 | 5.88 | 0.00 |
| Livestock | within | 11.49 | 76.12 | 48.09 | 44.65 | 84.31 | 0.00 |
| leopard cat | near | 0.00 | 18.18 | 3.70 | 2.45 | 2.94 | 0.00 |
| human | near | 268.56 | 1786.36 | 505.09 | 551.05 | 277.06 | 472.73 |
| Dog | near | 7.42 | 9.09 | 18.98 | 16.43 | 7.06 | 9.09 |
| Livestock | near | 149.78 | 118.18 | 46.76 | 73.08 | 146.47 | 109.09 |
| leopard cat | away | 0.00 | 0.00 | 3.57 | 4.72 | 1.23 | 1.24 |
| human | away | 206.02 | 0.00 | 43.97 | 95.59 | 26.99 | 42.56 |
| Dog | away | 4.82 | 0.00 | 2.70 | 2.52 | 2.15 | 5.37 |
| Livestock | away | 24.10 | 0.00 | 23.24 | 13.39 | 26.38 | 45.45 |
